# Self‐Healing Hydrogels: The Next Paradigm Shift in Tissue Engineering?

**DOI:** 10.1002/advs.201801664

**Published:** 2019-06-14

**Authors:** Sepehr Talebian, Mehdi Mehrali, Nayere Taebnia, Cristian Pablo Pennisi, Firoz Babu Kadumudi, Javad Foroughi, Masoud Hasany, Mehdi Nikkhah, Mohsen Akbari, Gorka Orive, Alireza Dolatshahi‐Pirouz

**Affiliations:** ^1^ Intelligent Polymer Research Institute ARC Centre of Excellence for Electromaterials Science AIIM Facility University of Wollongong NSW 2522 Australia; ^2^ Illawarra Health and Medical Research Institute University of Wollongong Wollongong NSW 2522 Australia; ^3^ DTU Nanotech Center for Intestinal Absorption and Transport of Biopharmaceuticals Technical University of Denmark Lyngby 2800 Kgs Denmark; ^4^ Laboratory for Stem Cell Research Department of Health Science and Technology Aalborg University Fredrik Bajers vej 3B 9220 Aalborg Denmark; ^5^ School of Biological Health and Systems Engineering (SBHSE) Arizona State University Tempe AZ 85287 USA; ^6^ Laboratory for Innovations in MicroEngineering (LiME) Department of Mechanical Engineering University of Victoria Victoria BC V8P 5C2 Canada; ^7^ Center for Biomedical Research University of Victoria 3800 Victoria Canada; ^8^ Center for Advanced Materials and Related Technologies University of Victoria 3800 Victoria Canada; ^9^ NanoBioCel Group Laboratory of Pharmaceutics School of Pharmacy University of the Basque Country UPV/EHU Paseo de la Universidad 7 01006 Vitoria‐Gasteiz Spain; ^10^ Biomedical Research Networking Centre in Bioengineering Biomaterials, and Nanomedicine (CIBER‐BBN) Vitoria‐Gasteiz 28029 Spain; ^11^ University Institute for Regenerative Medicine and Oral Implantology – UIRMI (UPV/EHU‐Fundación Eduardo Anitua) Vitoria 01007 Spain; ^12^ BTI Biotechnology Institute Vitoria 01007 Spain; ^13^ Department of Dentistry‐Regenerative Biomaterials Radboud University Medical Center Philips van Leydenlaan 25 Nijmegen 6525 EX The Netherlands

**Keywords:** cyborganics, nanocomposite hydrogels, nanomaterials, self‐healing hydrogels, tissue engineering

## Abstract

Given their durability and long‐term stability, self‐healable hydrogels have, in the past few years, emerged as promising replacements for the many brittle hydrogels currently being used in preclinical or clinical trials. To this end, the incompatibility between hydrogel toughness and rapid self‐healing remains unaddressed, and therefore most of the self‐healable hydrogels still face serious challenges within the dynamic and mechanically demanding environment of human organs/tissues. Furthermore, depending on the target tissue, the self‐healing hydrogels must comply with a wide range of properties including electrical, biological, and mechanical. Notably, the incorporation of nanomaterials into double‐network hydrogels is showing great promise as a feasible way to generate self‐healable hydrogels with the above‐mentioned attributes. Here, the recent progress in the development of multifunctional and self‐healable hydrogels for various tissue engineering applications is discussed in detail. Their potential applications within the rapidly expanding areas of bioelectronic hydrogels, cyborganics, and soft robotics are further highlighted.

## 1 Introduction

In recent years, tissue engineering has emerged as a promising technology to grow organs from scratch,^1^, ^2^, ^3^ replicate biological mechanisms of various diseases,^4^, ^5^, ^6^, ^7^ address tissue‐related ailments^8^, ^9^, ^10^, ^11^, ^12^ and enable life extension in the growing aging population.^13^, ^14^ So far, most of the tissue engineering approaches has relied on the encapsulation of stem cells within native‐like and highly porous biomaterials;^15^, ^16^, ^17^, ^18^, ^19^, ^20^, ^21^, ^22^ or scaffolds as the tissue engineers prefer to say. The scaffold‐based biomaterials enable encapsulated cells to spread and reorganize into tissue‐like architectures, while permitting sufficient nutrient and waste material exchange with the surrounding environment.

Of the many scaffolding biomaterials currently utilized for tissue engineering applications, hydrogels are among the most promising ones. Hydrogels are composed of polymeric networks that are capable of absorbing and retaining high amount of water.^19^, ^23^ Hydrogels are also tunable (both physically and chemically), are injectable, and have been used over the years for tissue engineering and various drug delivery applications.^24^, ^25^, ^26^, ^27^, ^28^ However, as one of the fascinating properties of natural tissues is their ability to self‐heal after minor injuries, to truly recapitulate the physical properties of native tissues, such human‐made biomaterials also need to spontaneously heal and regenerate injuries inflicted on them. This inherent ability of native tissues to regenerate on demand has initiated enormous motivation to develop intelligent hydrogels with similar self‐repair mechanisms.

**Figure 1:**
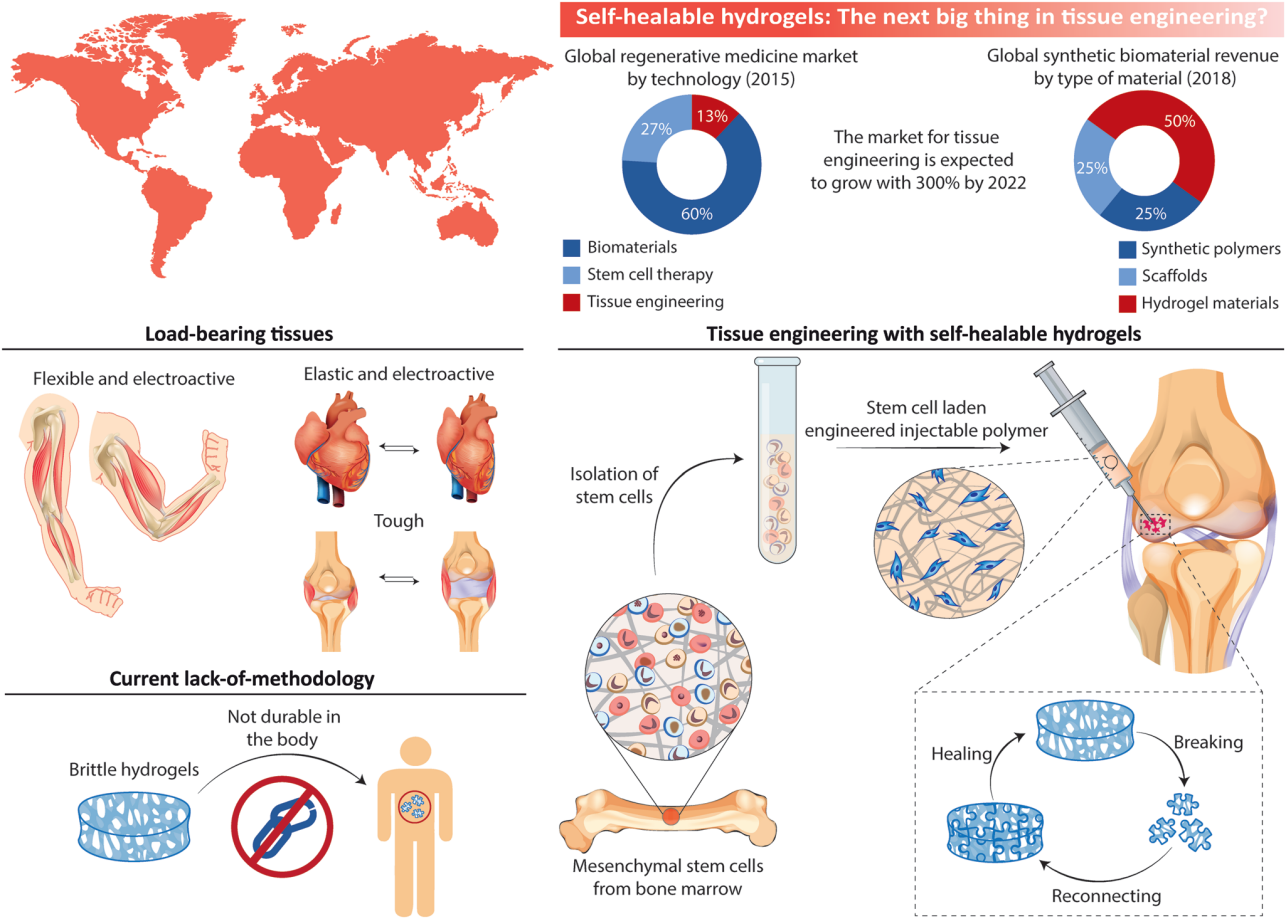
Human organs are made of elastic, tough, and electroactive tissues, which can spontaneously heal. The microenvironment within most tissues is also highly dynamic and load‐bearing. Tissue engineering hydrogels, therefore, need to heal on command and incorporate the same mechanical and electrical properties as those found in natural tissues. Injectability is also a sought‐after property, as injectable hydrogels can be used to deliver stem cells to the target tissue in a minimally invasive manner.

In spite of their many similarities to the extracellular matrix (ECM) of the native tissues, self‐healable hydrogels still face several shortcomings, which limits their specific application for replacement of electrically active and elastic tissues (**Figure** **1**).^29^, ^30^, ^31^, ^32^ For example, current self‐healable hydrogels are typically nonconductive and exhibit significantly lower fracture energies (<10 J m^−2^)^33^ than that of cartilage,^33^ skin,^34^ tendon,^35^ and muscle tissues^36^ (kJ m^−2^ regime). Conventional hydrogels implanted within the load‐bearing and dynamic environments of the human body are thus inclined to acquire some minor defects. These microcracks gradually propagate and grow in size and will ultimately lead to failure of the material if they are not repaired in due time. Moreover, in case of cell‐encapsulated hydrogels, cells are prone to rapid migration and interface pulling, and will eventually disrupt the structural integrity of the hydrogel matrix due to traction forces. Therefore, to achieve optimal implant lifetime, it will be necessary to engineer mechanically tough hydrogels with the ability to quickly remedy material defects.^37^, ^38^, ^39^, ^40^, ^41^, ^42^, ^43^

**Figure 2:**
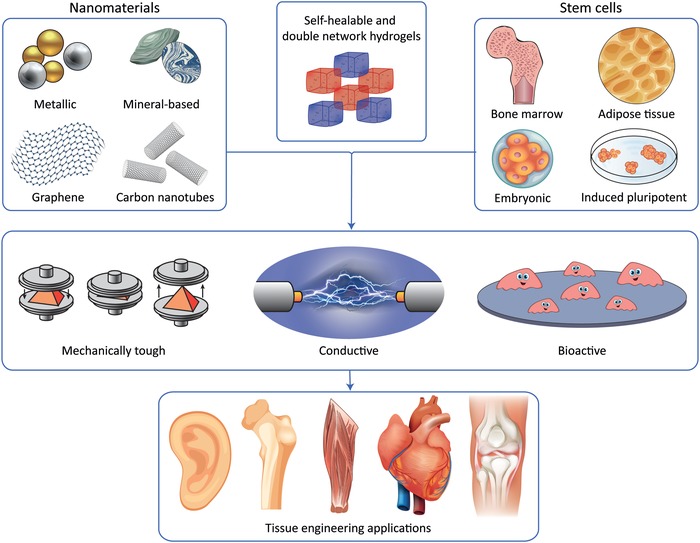
Nanoreinforcement can be used to generate multifunctional hydrogels that blend in with electrically and mechanically active tissues. With the right combination of nanoreinforcer and hydrogel polymer, it is possible to manufacture mechanically tough, electrically conductive, and bioactive tissue engineering systems for organ regeneration.

Although the literature on self‐healing hydrogels is growing fast, only a few practical applications for these biomaterials exist in tissue engineering; this is because most self‐healable hydrogels do not match with the above‐described electromechanical milieu of the body (Figure 1). Moreover, the long‐standing incompatibility between hydrogel toughness and rapid self‐repair has not yet been fully addressed. To address this unmet need, nanomaterials are rapidly emerging as an exciting approach to develop self‐healable and multifunctional hydrogels through one‐step strategies that are based on simple mixing procedures (**Figure** **2**).

In this review article, we discuss the unexplored and enormous possibilities of self‐healable hydrogels in tissue/organ regeneration and repair. We will mainly focus on the synergy between hydrogel toughness and dynamic self‐repair, as we believe this unique combination will ultimately induce a paradigm shift in the field of hydrogel‐based tissue engineering. Additionally, we will also highlight the emerging area of self‐healable hydrogels made through nanoreinforcement and review the many recent impressive applications of these systems in tissue engineering. Finally, we will review the application of self‐healable hydrogels in cyborganics, soft robotics, and bioelectronics, since these fields will rise in the coming decades and define an entire new frontier in health sciences.

## 2 Self‐Healing mechanisms

**Figure 3:**
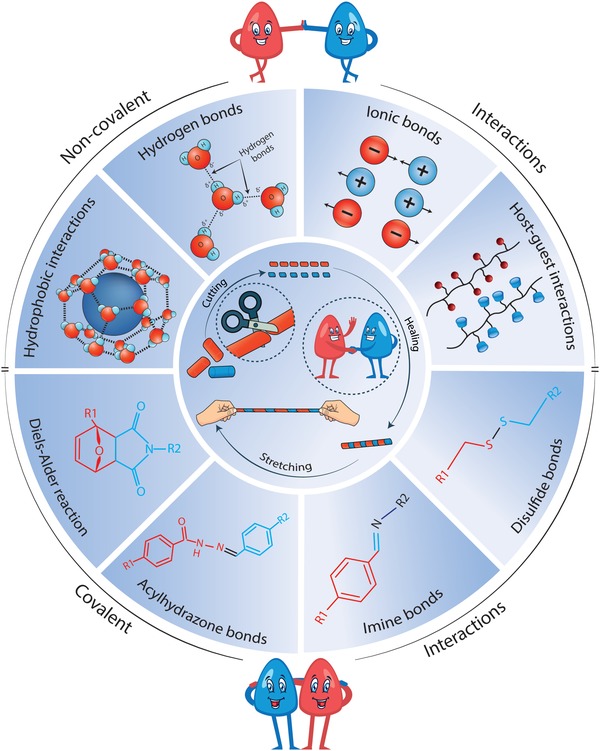
The various chemical and noncovalent interactions behind self‐healable hydrogels are highlighted here.

Self‐healable hydrogels rely on one common principle involving a so‐called mobile phase, which enables crack closure through a combination of mass transfer and reconnection of broken links within the hydrogel matrix. The reconnection within this matrix is typically mediated by either noncovalent or covalent bonds (**Figure** **3**). The noncovalent interactions are based on weak sacrificial links such as ionic,^44^ hydrogen,^45^ or hydrophobic bonds,^46^ while the chemical bonds are based on dynamic covalent bonds and metal coordination bonds.^47^, ^48^, ^49^, ^50^, ^51^, ^52^ The combination of the above‐mentioned covalent and noncovalent bonds has also recently been used to generate mechanically tough and elastic double‐network hydrogels with rapid self‐repair properties.^53^, ^54^, ^55^ In this section, we will highlight the key mechanisms behind the repairing properties of such self‐healable hydrogels (Figure 3). Specifically, we have divided this section into three independent subsections, the first two dealing with self‐healing mechanisms based on either noncovalent or covalent bonds, and the last section on the working principle behind double‐network hydrogels.

### 2.1 Noncovalent Interactions

This section focuses on self‐healable hydrogels made from noncovalent crosslinks including hydrogen bonds,^53^, ^56^, ^57^, ^58^, ^59^, ^60^ host–guest interactions,^61^, ^62^, ^63^ ionic bonds,^64^ and hydrophobic interactions.^65^, ^66^, ^67^ Although the listed interactions can generate hydrogels that display rapid healing time and good self‐healing efficiency, these attributes come at the cost of inelasticity and mechanical weakness. In the following subsections, we describe the pros and cons of each of these interaction schemes from a mechanical‐point‐of‐view and provide possible solutions that could remedy their current shortcomings.

### 2.2 Hydrogen Bonds

Hydrogen bonds are among the most common noncovalent interactions found in nature. For instance, it is hydrogen bonds that keep complementary strands of DNA together in their unique helix structure,^68^ and they are also a key player in water's many solvent properties (Figure 3).^69^ They also play a determining role in the secondary and tertiary structure of folded proteins^70^ and the properties of various solid polymers such as cellulose and wool.^71^, ^72^

Hydrogen bonds occur when the positive hydrogen atom establishes an electrostatic link with electronegative acceptor atoms such as oxygen, nitrogen, or fluoride.^73^ The strength of the hydrogen bonds depends mostly on the negative charge of the acceptor atom and can thus vary greatly depending on the atom in question and the pH value of the solvent in which the bonding occurs. Examples of hydrogen bonds are those formed between two hydroxyl groups (OH···OH), between carboxyl and amide groups (NH···O=C), and between hydrogen and fluorine (H···F). The bonding strength of hydrogen bonds varies greatly, from 0.25 to 15 kcal mol^−1^, with the weakest being those formed with fluorine and the strongest those that incorporate hydroxyl or amide groups.^73^

Even though a hydrogen bond is several times weaker than ionic (100–1000 kcal mol^−1^) and covalent bonds (35–240 kcal mol^−1^),^74^ they still contribute significantly to the mechanical properties of hydrogels, when a lot of them are present within the hydrogel matrix. Moreover, the association and dissociation of hydrogen bonds occur rapidly; typically on sub‐picosecond to picosecond time scales.^75^, ^76^, ^77^ This property is a governing factor behind the rapid healing time seen in repairable hydrogels based on hydrogen bonds. For these reasons, hydrogen bonds have been exploited in the manufacture of numerous self‐healable hydrogels, the most prominent of these being those using poly(vinyl alcohol),^53^, ^56^ ureidopyrimidinone (UPy),^57^, ^58^, ^59^, ^60^ and polyacrylamide (PAM);^53^, ^78^ with the hydrogen bonds being mediated by either hydroxyl or amide groups. However, due to their weakness, hydrogen bonds need to be combined with other stronger bonds to yield self‐healable hydrogels with the desired combination of mechanical strength and fast healing time. We will return to cover this important point in Section 2.4., when discussing how double‐network hydrogels work and what they do.

#### 2.2.1 Hydrophobic Interactions

Hydrophobic interactions are perhaps as important as hydrogen bonds when it comes down to protein folding, the properties of solid polymers, and the interaction between molecules in different solvents.^79^ However, hydrophobic interactions are slightly stronger than hydrogen bonds and easier to control; as they can be fine‐tuned by varying the shape of the hydrophobes and the number of hydrophobic moieties on them. Hydrophobic interactions occur because of the formation of a clathrate cage around the hydrophobe, which is an ice‐like cage structure of water molecules formed through hydrogen bonds among the water molecules (Figure 3).^80^ This highly ordered arrangement leads to entropy decrease and constitutes an unstable configuration, which easily breaks when two hydrophobes come close enough to one another to enable the formation of hydrophobe–hydrophobe assemblies, and the subsequent release of trapped water between the two. The highly adhesive force between two hydrophobes is thus caused by a physically driven increase in entropy, which is also a governing factor behind the highly reversible nature of hydrophobic interactions.^79^

Some different hydrophobic schemes based on 1) host–guest interactions,^61^, ^62^, ^63^ 2) micelles,^81^, ^82^ and 3) hydrophobic moieties^65^, ^66^, ^67^ have been used over the years to generate self‐healable hydrogels. Self‐healable micelle‐based hydrogels are generated by incorporating amphiphilic polymers and surfactants into the hydrogel. The self‐healing mechanisms of such hydrogels are attributed to the cyclic dissociation and reassociation of the micelles. The self‐repair mechanisms underlying hydrogels that incorporate hydrophobic moieties, on the other hand, arise from reversible interactions between such moieties. Host–guest interactions are more complicated and, in most cases, are ruled by the conjugation of cyclodextrin—a molecule that consists of a lipophilic inner cavity and a hydrophilic outer surface—onto the hydrogel backbone.^61^ To this end, cyclodextrin can enable a so‐called host–guest interaction with hydrophobic guest molecules, as they can become restrained within its lipophilic inner cavity due to hydrophobic interactions. One important concern of the above‐mentioned self‐healable hydrogels is the possible low water‐uptake caused by the presence of hydrophobic regions within the hydrogel matrix. However, numerous studies have shown that this does not need to be the case when hydrophilic regions outbalance the number of hydrophobic moieties in the hydrogel matrix.^81^, ^83^, ^84^

#### 2.2.2 Ionic Bonds

As an alternative strategy, ionic bonds can also be used to develop self‐healable hydrogels via reversible electrostatic interactions between oppositely charged moieties. Such interactions can happen between oppositely charged polymers or through ionic bridges between same charged polymers mediated by oppositely charged ions.^85^, ^86^, ^87^ A common example of the later is alginate hydrogels made from negatively charged alginate pre‐polymers that crosslink into a hydrogel through divalent ions; typically calcium.^88^, ^89^ A less common but yet highly promising approach is to use electrostatic interactions between charged nanomaterials and the hydrogel backbone, as this approach can add a range of additional properties to the system in question because of the multifunctional attributes of nanomaterials.^8^, ^90^, ^91^, ^92^ For instance, nanomaterials are known for their ability to dissipate energy within hydrogels, and can, therefore, increase the toughness and durability of hydrogels significantly.^93^ Although ionically bonded hydrogels are simple, as they are manufactured by one‐step‐mixing procedures, their inelasticity and brittleness are major drawbacks. One avenue to overcome the shortcomings of ionically crosslinked hydrogels is blending them with a covalently cross‐linkable polymer to yield a double‐network hydrogel consisting of interpenetrating and adaptable polymeric networks.^94^, ^95^

### 2.3 Covalent Interactions

In addition to the noncovalent self‐healing mechanisms discussed in the previous section, covalent binding schemes in the form of chelation and dynamic covalent bonds can also be used to generate self‐healable hydrogels. In this section, we will focus on the chemistry of these with special focus on dynamic covalent bonds made from imine, disulfide, boronate ester, acylhydrazone, and Diels–Alder reactions (Figure 3).

#### 2.3.1 Chelation

Chelation can be described as a number of coordinate bonds between ligands (organic molecules) and one positively charged transition‐metal ion.^96^ Notably, the transition metal ion is surrounded by the ligands to yield highly complex lattice structures. Each ligand can donate electrons to the metal ion; two or more electrons from each ligand are typically donated to the metal. The bonding is thus essentially a covalent bond involving two electrons from the same atom instead of one electron from each atom, which is the typical case in standard covalent bonds. However, due to the lattice structure of these metal complexes and the many donor atoms involved, the binding energy of the complexes is typically stronger than covalent bonds.^97^, ^98^ But what makes chelation unique in comparison to covalent bonds is the fact that they can display high adhesivity, elasticity, and reversibility at the same time. For this reason, chelation can be used to yield highly adhesive, elastic, and self‐healable materials. A prime example of this can be found in nature in the form of the sticky feet of mussels, which in recent years have been linked to chelation between Fe^+3^ and catechol ligands.^99^ Indeed, the binding strength of catechol‐Fe^+3^ can reach 33 kcal mol^−1^ and catechol can form reversible bonds with titanium interfaces with a bond strength of 800 pN, which is almost 40% of the bonding strength between silicon and carbon.^100^ This gives an idea of some of the incredible features of chelation complexes, and why they have constituted an integral component in many self‐healable hydrogels.^48^, ^101^, ^102^

#### 2.3.2 Dynamic Covalent Bonds

Dynamic covalent bonds are unique because of their ability to reconnect without physical stimuli, which is in contrast to standard covalent bonds. Dynamic covalent bonds, therefore, combine the stability of covalent bonds and reversibility of noncovalent interactions into a potent healing force that works entirely on its own. The general principle behind such dynamic bonds is the presence of an equilibrium phase between various fates from the same reaction process. Under certain conditions, one of the fates is more stable and dominates over the others. However, a return to the original compounds followed by a reversion to another outcome is still possible, which makes such reactions highly dynamic.^103^, ^104^ As such, these are highly sought out in the field of chemistry, but their numbers are relatively few compared to conventional covalent reactions. Nevertheless, various self‐healable hydrogels have been produced over the years through dynamic covalent crosslinks, with imine bonds being the most widely used. Thus, we will first focus on the chemistry of these bonds and their usefulness for self‐healing.

The famous German chemist, Hugo Schiff, discovered imine bonds in 1864 and imine‐based compounds are therefore also commonly referred to as “Schiff's bases.”^105^ An imine bond essentially involves a reaction between an aldehyde and a primary amine with the generation of a water molecule; and is considered a strong covalent bond (150 kcal mol^−1^)^106^ that can occur both at neutral and acidic pH values. In this reaction, the amine nitrogen (a nucleophile) attacks the electrophile carbonyl atom in the aldehyde to yield a double nitrogen–carbon bond. If the water molecule is not removed, this reaction can still go back through hydrolysis and, therefore, under certain conditions, a dynamic equilibrium is possible. Since imine bonds involve an amine group, they are frequently used to turn amino‐rich polymers, such as chitosan and polyacrylamide, into self‐healable hydrogels by combining them with other aldehyde‐functionalized polymers, such as oxidized alginate and hyaluronic acid (HA), as described in Section 3.^50^, ^107^

Acylhydrazone bonds are very close relatives of imine bonds, as they are synthesized by reacting a hydrazine with an aldehyde group;^108^, ^109^ typically through a condensation reaction.^50^, ^110^ Acylhydrazone bonds can be spontaneously formed under physiological conditions; albeit at a significantly slower rate than under acidic environments.^111^, ^112^ However, recent studies have shown that this bonding scheme can be used to yield self‐healable hydrogels with a crosslinking time that makes them amenable as injectable hydrogel carriers for stem cells.^50^, ^113^ In one of these studies, a self‐healable polyethylene glycol (PEG) hydrogel was developed through a condensation reaction between two PEG macromers; one functionalized with benzaldehyde and the other with an aldehyde. This system could self‐assemble rapidly under physiological conditions and provided a viable environment for encapsulated muscle cells.^113^

Another dynamic covalent bond type used in self‐healable hydrogels, although a bit weaker than imine bonds (50 kcal mol^−1^),^106^ is disulfide bonds. They are essentially based on thiol/disulfide dynamic exchange reactions, in which the thiol groups needs to be oxidized.^114^, ^115^ The reaction is, therefore, highly sensitive to the pH value and needs the involvement of an oxidation agent, which can make some of the manufacturing protocols for such hydrogels cytotoxic for cells. For these reasons, thiol‐based hydrogels with self‐healing capacity are not stable in physiological tissues due to the presence of reducing agents such as glutathione, which is found in most tissues in the body.^116^, ^117^, ^118^ These are, therefore, not the preferred choice for the design and development of self‐healable hydrogels.

The combination of diols and boronic acid can also yield reversible covalent links in the form of boronate esters.^52^, ^119^ However, the stability of this reaction is highly pH sensitive and the resultant self‐healing efficiency and mechanical properties of such systems are therefore sensitive to pH changes. Indeed, the formation of diol‐boronic acid links only happens at pH values greater than or equal to the pKa value of boronic acid; which is typically greater than 8 pKa.^120^, ^121^ From a tissue engineer's point‐of‐view, this can be a disadvantage, as most tissues in the human body operate at neutral pH and the fact that cells perish at pH values above 8. However, in a recent study, it was shown that the combination of PEG–phenylboronic acid macromonomers with PEG–diol macromonomers could yield injectable hydrogels with sufficient self‐healing efficiency and mechanical properties at neutral pH.^122^ In another recent study, a self‐healing hydrogel was formed using intramolecular interactions between 2‐acrylamidophenylboronic acid (2APBA) moieties. This hydrogel could self‐heal at both neutral and acidic pH values and is thus suitable for tissue engineering applications.^51^ These studies, taken together, have demonstrated that, despite the highly pH‐sensitive nature of boronate esters, they can be used to develop self‐healable hydrogels that are compatible with tissues and cells.

In addition to the above‐mentioned reactions, Diels–Alder reactions have also been rapidly adopted by scientists in the field to yield self‐healable hydrogels. Diels–Alder reactions are considered click reactions, which are widely recognized for their ability to yield outstanding reaction specificity through simple synthesis procedures typically done in water and with no offensive byproducts.^123^ In simple terms, a Diels–Alder reaction is a reaction between a conjugated diene and a dienophile, typically an alkene or alkyne.^124^ Such reactions are essentially electrocyclic reactions that involve π electrons from the HOMO and LUMO molecular orbitals of diene and the dienophile, respectively. To this end, to enable the reaction to proceed optimally, it is important that there is an energy‐band overlap between the two molecular orbitals.^124^ Diels–Alder reactions are also thermoreversible, meaning that they break at elevated temperatures—typically above 100 °C—and can reform again once the temperature is lowered. This process is cyclic and, therefore, enables the manufacture of materials that can break and heal indefinitely by applying the appropriate thermal healing procedure.^125^, ^126^, ^127^ This is interesting but, unfortunately, also implies that the self‐healing potential of conventional Diels–Alder bonds is not fit for use in tissue engineering. However, some recent studies have shown that it is possible to generate Diels–Alder‐based self‐healable hydrogels that can solidify and self‐heal under physiological conditions.^128^, ^129^ The family of Diels–Alder reactions, therefore, has promise for delivering injectable and self‐healable hydrogels that can crosslink autonomously within the target tissue, if prepared correctly.

### 2.4 Double‐Network Hydrogels

An on‐going challenge in the design and development of hydrogels is the conflict between strength, toughness, and high water content (>90 wt%). Given that strong materials tend to be brittle, softer materials usually tougher, and highly hydrated materials weak, this challenge is difficult to overcome.^130^, ^131^ Another challenge in the field is to combine fast self‐healing kinetics with hydrogel strength, as mechanically strong hydrogels are typically achieved by increasing the polymer concentration and the number of crosslinks within the hydrogel matrix. The latter, unfortunately, reduces the mass transfer into the crack site and, therefore, significantly increases the healing time.

**Figure 4:**
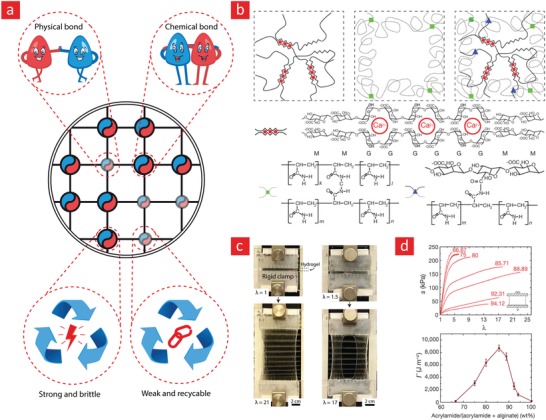
A brief depiction of the concept behind double‐network hydrogels and what they can do. The working principle behind double‐network hydrogels is essentially based on a combination of weak reversible bonds and strong irreversible bonds. Together these can yield tough and highly extendible hydrogels due to the dissipation mechanism embedded in the reversible bonds. a) A prime example of systems like this was given in a recent publication in nature letter that was based on b) ionically bonded alginate (reversible) and covalently bonded polyacrylamide (irreversible) polymers. c) This hydrogel could stretch up to 21‐times its original length and d) exhibited a toughness value around 10 000 J m^−2^. Reproduced with permission.^94^ Copyright 2012, Macmillan Publishers Ltd.

Double‐network hydrogels address all of these challenges by combining a strong and rigid network with a much weaker network that is typically made from reversible crosslinks (**Figure** **4**).^94^, ^95^, ^132^, ^133^, ^134^ Hydrogels made in this manner have demonstrated some amazing properties, including high elastic modulus (0.1–1.0 MPa), strength (1–10 MPa), stretchability (1000–2000%), and toughness (100–10 000 J m^−2^).^94^, ^132^ Notably, double‐network hydrogels can be used to develop mechanically strong hydrogels with rapid self‐healing properties using much lower polymer concentrations than their single‐network counterparts.^63^, ^102^ Indeed, the mechanical properties that double‐network hydrogels display are highly sought‐out attributes in the field, as they resemble those of conventional rubbers (1000 J m^−2^) and load‐bearing cartilage tissues (100–9000 J m^−2^);^94^, ^132^ while being tougher than commercially available hydrogels, which are notoriously weak and brittle (0.1–10 J m^−2^) and, therefore, unable to resist the cyclic forces that tissues such as muscle, bone, heart, and cartilage endure during daily routine activities.^94^, ^132^

The toughening mechanism of double‐network hydrogels stems from the presence of strong irreversible and weak reversible bonds within the hydrogel matrix. The weak bonds can reform again and thus limits the amount of stress accumulation within the matrix, as these bonds can undergo many destruction–reconnection cycles. In simple terms, one can therefore attribute the amazing mechanical properties of double‐network hydrogels to a rigid skeleton that keeps the system intact, and sacrificial bonds that enable energy dissipation, delaying the onset of critical stress accumulation.^94^, ^132^, ^133^, ^134^ This mechanism is similar to the mechanism used by the body to toughen bone,^135^ and by engineers to generate structural materials that are both strong and tough at the same time.^130^

In a recent study, a double‐network hydrogel made from a covalently crosslinked polyacrylamide and ionically crosslinked alginate network displayed some interesting properties, as this system could stretch up to 2000% of its original length with a toughness value that could reach 9000 J m^−2^ (Figure 4).^94^ The authors speculated that the reversible ionic bonds enabled an energy dissipation scheme that could keep going as the hydrogel stretched, leading to the reported combination of high toughness, strength, and elasticity. The working principle behind double‐network hydrogels therefore presents an exciting avenue to remedy the inherent mechanical weakness of hydrogen‐bonded self‐healable hydrogels;^53^, ^136^, ^137^ and as we will see in Sections 3 and 4, this combination can generate self‐healable hydrogels that could transform the world of tissue engineering if they can be translated into the clinic.

### 2.5 Outlook and Future Opportunities

In conclusion, a wide selection of reversible bonds is currently available for the design and development of self‐healable hydrogels. However, neither the physical nor the chemical bonds can, on their own, address the many requirements for self‐healable tissue engineering hydrogels. These requirements include high toughness, mechanical strength, high water content, injectability, elasticity, and fast self‐healing kinetics. Nevertheless, the combination of physical and chemical bonds in the form of double‐network hydrogels can address this current lack‐of‐methodology in the field, as evident from recent publications in Nature Letters^94^ and Nature Materials.^134^ In the authors' opinion, this area of research is still ripe for investigation, and a successful outcome to this end could yield the next gold standard in implantable materials for tissue engineering applications. One avenue that might open the field even further is the development of a double‐network hydrogel with well‐defined matrix architecture. Indeed, some recent studies have shown that by aligning polymers^138^, ^139^ or nanomaterials^140^, ^141^ within hydrogels, it is possible to significantly improve mechanical integrity without increasing the polymer concentration or the crosslinking density. We, therefore, anticipate that this unique combination can result in self‐healable hydrogels with mechanical properties that might match that of skeletal tissues, without compromising the self‐healing or water retention properties. On a more fundamental level, we envision that other dynamic covalent bonds could be used to further improve the state‐of‐the‐art in the emerging area of self‐healable tissue engineering hydrogels. For instance, dynamic covalent bonds, such as those based on amide and ester exchange reactions,^142^ could—together with weak hydrogen bonds—yield some exciting double‐network hydrogels for the field.

## 3 Polymers for Self‐Healing Hydrogels

One of the most important components in hydrogels, are polymers, that can be united into water friendly 3D environments for cells to attach, grow, and differentiate within.^143^ The logical way to obtain self‐healing hydrogels is, therefore, by altering these essential building blocks. This is typically accomplished by incorporating the aforementioned self‐healing mechanisms into the polymeric hydrogel backbone through various nontoxic and nonhazardous chemical modifications.

The healing capacity of polymeric hydrogels is typically accessed by monitoring the rejoining of two broken pieces by either optical or scanning electron microscopy (SEM). Another more precise quantification of the healing capacity includes mechanical characterization of the ultimate tensile strength and Young's modulus of the hydrogels before and after the healing process. The healing efficiency is then defined as the ratio of the modulus or tensile strength of the healed and unbroken hydrogels. Finally, it is also possible to use rheology to gain a detailed picture of both the healing efficiency and healing time by monitoring the real‐time changes in storage and loss modulus during the healing process.

**Figure 5:**
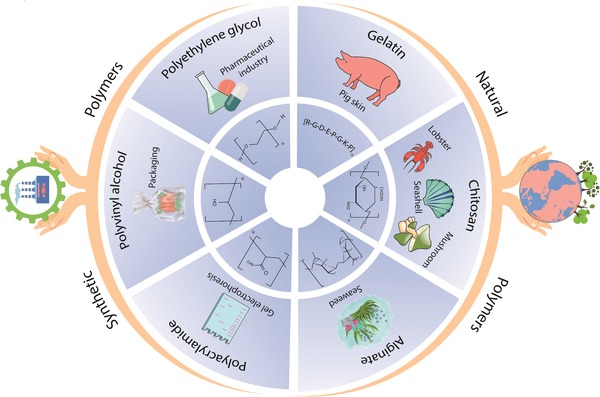
The various synthetic and natural polymers used to generate self‐healable hydrogels are highlighted here.

Self‐healing hydrogels have so far been made from either natural or synthetic polymers (**Figure** **5**). The natural polymers are for the most part polysaccharide‐based polymers, such as alginate, chitosan, and HA. The synthetic polymer systems, on the other hand, are based on polymers such as polyethylene glycol, poly(acrylic acid), poly(vinyl alcohol), and polyacrylamide. These polymers provide certain advantages and disadvantages and abandoning one in favor of another typically involves some trade‐offs. For example, the natural polymers are typically more biocompatible, as polymers such as gelatin and chitosan are famed for their cell attachment properties,^144^, ^145^, ^146^, ^147^, ^148^ while poly(vinyl alcohol) and polyacrylamide typically yield stronger and more elastic hydrogels at the cost of reduced biofriendliness. In the following subsections, we will review these polymers as potential candidates for self‐healing hydrogels in the field of tissue engineering.

### 3.1 Natural Polymers

Over the centuries, natural materials have been a great source of inspiration for materials scientists and physicians; noteworthy examples in this regard are silk‐based sutures^149^ and cellulose‐based building materials.^150^, ^151^ In recent years, biomedical engineers have also started to tap into natural sources to develop even better biomaterials. Because one of the prime‐ingredients in native tissues is HA—a polysaccharide—the focus has for the most part been directed toward polysaccharides, such as alginate, pectin, and chitosan, which can be procured from natural sources. These naturally derived polymers are cheaper than HA and less immunogenic than, for instance, gelatin. The focus of this section is, therefore, directed toward alginate and chitosan‐based self‐healable hydrogels, however, we will also briefly highlight some of the emerging trends in self‐healable hydrogels made from gelatin and HA.

#### 3.1.1 Alginate

Alginates are naturally occurring polysaccharides typically retrieved from marine algae and brown seaweed.^152^ Because of their good biocompatibility, exceptional water retention, and tunable gelation properties, alginates have been extensively studied as soft scaffolds in various tissue engineering applications, and as microcapsules for delivery of drugs.^153^, ^154^, ^155^, ^156^, ^157^ However, alginate‐based hydrogels are brittle and mechanically unstable, which significantly limits their use in many biomaterial applications.^89^ One approach to remedy this is by making self‐healing alginate hydrogels with the capacity to spontaneously self‐repair in the event of mechanical damages.

Recently, a variety of methods have been applied to generate self‐healing alginate hydrogels; something that has enticed much attention in the field of biomaterial science.^50^, ^158^, ^159^ These methods include various chemical methods to incorporate dynamical covalent bonds into the polymeric backbone of alginate^50^, ^159^ and through the concept of host–guest interactions.^158^ For instance, in a recent study, the noncovalent interaction between a host [β‐cyclodextrin grafted alginate (alg‐*g*‐CD)] and a guest (Pluronic F108) was shown to facilitate a fast healing performance alongside desirable thermoresponsive gelling properties and negligible cytotoxicity.^158^ As alginates contain functional hydroxyl groups, which are transformable into aldehyde groups, dynamical imine bonds arising from the interaction between aldehyde groups and amines have also become a central theme in the development of self‐healable alginate hydrogels.^50^, ^159^ As an example of this feasibility, Wei et al. used chitosan (amine rich) and oxidized alginate (aldehyde rich) to yield a mechanically robust hydrogel with excellent self‐healing ability (up to 95% healing efficiency) and good cytocompatibility, as tested through encapsulation with 3T3 fibroblast cells.^50^ The hydrogel developed in this study was an engineering masterpiece as it achieved the rare union between high self‐healing efficiency and high mechanical strength, while still keeping the hydrogel biocompatibility intact. Such hydrogels will assist scientists to carve new avenues in the field of tissue engineering, which we anticipate will enable the manufacture of scaffolds with the ability to integrate with native tissue over sustainable periods. Along the same line, a hydrogel composed of dopamine‐grafted oxidized sodium alginate (OSA‐DA) and PAM, showed efficient self‐healing ability (80% mechanical recovery in 6 h), high tensile strength (0.109 MPa), and ultrastretchability (2550%). Remarkably, due to plenty of catechol groups on the OSA‐DA chains, the hydrogel possessed unique cell affinity (NIH‐3T3 fibroblasts) and tissue adhesiveness. Furthermore, the in vivo rat experiment showed that this hydrogel could promote tissue regeneration and accelerate the process of wound healing.^160^

Quite often multicomponents are utilized to construct hydrogels with self‐healing ability and the resulting hydrogel systems often exhibit batch‐to‐batch dependent inconsistent results, problems in multicomponent mixings, and involve unpredictable cross‐talk between the added components. To this end, Hong et al., developed a “single polymeric component,” based on alginate‐boronic acid (alginate‐BA) to overcome the aforementioned problems.^161^ This stretchable hydrogel showed efficient self‐healing performance (up to 98% healing efficiency), owing to reversible inter‐ and intramolecular interactions. In addition, subcutaneous implantation of this hydrogel under mice skin showed inflammatory response at day 3 which mostly disappeared on day 7, indicating the low toxicity of the hydrogel.

#### 3.1.2 Chitosan

**Figure 6:**
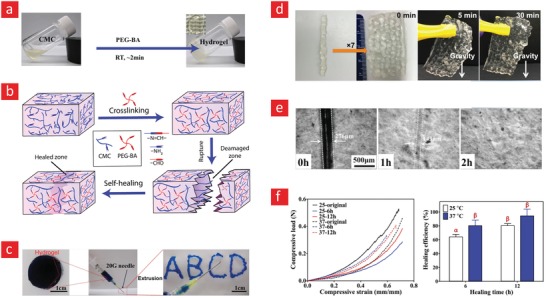
A mechanically strong and self‐healable carboxymethyl–chitosan (CMC) hydrogel based on Shiff‐base bonds. a) The gel formation is shown here along with b) the chemical mechanism behind its self‐healing properties. c,d) The self‐healing properties of the CMC hydrogel. e) The mechanical properties of the as‐prepared hydrogel and those that were allowed to heal for 6 and 12 h. f) The healing efficiencies (the compressive load ratio of as‐prepared and healed samples) at 25 °C and 37 °C are shown here; there were no significant differences between bars with the same letters. Adapted with permission.^43^ Copyright 2016, Wiley‐VCH.

Chitosan is a positively charged and amino‐rich polysaccharide typically derived from the exoskeleton of shellfish and possesses similar structural characteristics as glycosaminoglycan (GAG); one of the major components of the ECM.^162^, ^163^ Due to its similarities with the native ECM, chitosan is also biocompatible, biodegradable, and hydrophilic, which makes it an ideal 3D microenvironment for cell encapsulation. For these reasons, chitosan has frequently been used as a scaffolding material for tissue engineering^144^, ^164^, ^165^ and various cell delivery therapies.^166^, ^167^, ^168^ As chitosan is rich in amino groups, the ideal pathway for generating self‐healing hydrogels with chitosan is through dynamic covalent imine/enamine bonds.^43^, ^50^, ^107^, ^169^, ^170^ To this end, a recent study by Huang et al. used an enamine bonding between carboxymethyl chitosan (CMC) and an aldehyde functionalized polyethylene glycol polymer (PEG‐BA) to develop a self‐healing hydrogel (**Figure** **6**).^43^ The two polymers formed a stable hydrogel film after 5 min with a good storage modulus (3.2 kPa) and a healing efficiency ranging from 80% to 94% at physiological temperature (37 °C), depending on the healing time (6–12 h). Encapsulation studies with fibroblast cells demonstrated excellent cell viability for up to 7 d, indicative of chitosan's potential as a regenerative milieu for tissue engineering applications. In a similar vein, chitosan and cellulose acetoacetate were mixed to endow a hydrogel with good self‐healing ability on account of dynamic enamine bonding between aldehyde groups of the functionalized cellulose and amino groups of chitosan.^169^ Specifically, the broken gels were able to heal after 40 min at 37 °C in physiological conditions. Despite increasing usage of chitosan in self‐healing hydrogels, low water solubility of this polymer at physiological pH can be problematic for its application in tissue engineering. To this end, Khan et al. synthesized a water‐soluble derivative of chitosan by grafting l‐glutamic acid onto its backbone (chit‐glu). Subsequent mixing with benzaldehyde‐terminated 4‐arm poly(ethylene glycol) (PEG‐BA) led to fast formation (<60 s) of a gel, based on imine bonds. The prepared hydrogels were shown to be injectable and self‐healing, and the healed hydrogels fully recovered their initial elastic modulus in a compression test. Most remarkably, human fibroblast cell lines (WI‐38) cultured on top of the hydrogel were viable and capable of proliferating.^171^

Noncovalent interactions based on hydrogen bonds^172^, ^173^ and hydrophobic forces^174^ have also been utilized to fabricate self‐healable chitosan hydrogels. For instance, a pH‐sensitive hydrogel made from hydrogen‐bonded chitosan (CS) and polyvinyl alcohol (PVA) was capable of completely healing itself after 1 h and showed good cytocompatibility with HeLa cells.^175^ In a similar study, a multilayered polyelectrolyte composite film comprised of CS and polyacrylic acid (PAA) was demonstrated to pose excellent self‐healing ability at low PH values.^172^ In another study, hydrophobic interactions were used to develop a chitosan‐based self‐healable hydrogel.^176^ Specifically, in this study, chitosan was modified with the hydrophobic compound—ferrocene—to enable self‐healing through hydrophobic interactions between adjacent ferrocene sites. This hydrogel was also highly stimuli‐responsive and could be triggered to deliver small molecules and biologics by changing the pH value. In the authors' opinion, stimuli‐responsive and self‐healable hydrogels are ideal for on‐demand delivery of tissue growth factors and stem cells to injury sites in the body and could potentially change the course of the field of tissue engineering.

#### 3.1.3 Hyaluronic Acid

HA is a nonsulfated glycosaminoglycan that is found in ECM of vertebrate tissues,^177^ and is a suitable tissue engineering polymer due to its ability to bind cell surface receptors such as CD44, as well as its involvement in regulating cell differentiation and proliferation.^178^ In addition, the presence of reactive carboxylic groups on HA enables various chemical functionalization schemes for further downstream applications.^179^ Nonetheless, similar to other hydrogels made from naturally derived polymers, HA‐based hydrogels are susceptible to mechanical disruption, and accordingly, a variety of methods have been applied to develop self‐healable and durable HA hydrogels. These methods include the use of chemical bonds such as Diels–Alder and acylhydrazone bonds^180^ or noncovalent interactions such as guest–host interactions^38^, ^181^, ^182^, ^183^, ^184^ and hydrogen bonds.^45^ For instance, a recent study has used a combination of Diels–Alder and acylhydrazone bonds to make a self‐healing hydrogel (healing after 3 h) with good mechanical property (storage modulus of 18 kPa) from a mixture of oxidized furan–HA, furan–adipic dihydrazide‐functionalized HA, and dimaleimide–PEG.^180^ Specifically, Diels–Alder click chemistry between furan and maleimide was utilized to give the hydrogel matrix its mechanical integrity, while the self‐healing mechanism was built into the system via reversible acylhydrazone bonds between acylhydrazine and the aldehyde groups present on the oxidized furan–HA backbone. Most remarkably, this hydrogel showed great adhesion to native cartilage tissue (adhesive strength of ≈10 kPa), owing to the aldehyde‐amine Schiff‐base reaction. Based on a similar principle, a hydrogel composed of hydrazide‐modified HA (HA‐HYD) and aldehyde‐modified HA (HA‐ALD), exhibited fast self‐healing (healing after 10 min) with 100% healing efficiency.^185^ Additionally, 3T3 fibroblast cells encapsulated within the hydrogel demonstrated high cell viability (after 14 d of culture) in accordance with a high biocompatibility.

**Figure 7:**
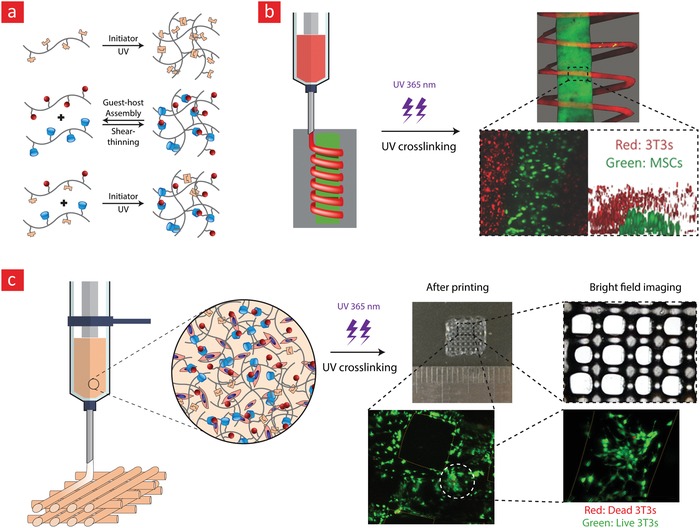
A self‐healable and printable HA‐hydrogel based on host‐guest interactions. a) The chemistry behind the self‐healing mechanism. b) A depiction of the cell‐laden hydrogel patterns with helical features, which were generated through extrusion printing. c) The team also used the extrusion printing technique to generate a grid‐like 3D construct consisting of 3T3 cells, which displayed high viability. Adapted with permission.^188^ Copyright 2016, American Chemical Society.

With regard to self‐healable hydrogels generated through physical crosslinking, a series of milestone studies from Jason Burdick's laboratory are worthy of mentioning.^38^, ^181^, ^182^, ^183^, ^184^ In most of these studies, guest–host interactions between adamantine(guest)–HA and β‐cyclodextrin(host)–HA were used to develop self‐healable hydrogels, which at the same time also were suitable for printing 3D scaffolds due to their shear‐thinning properties.^38^, ^182^, ^186^, ^187^, ^188^ In some of these studies, a methacrylated HA precursor polymer was added to the system for stabilizing the finalized scaffolds through UV‐crosslinking of the methacrylate groups (covalent bonding).^38^, ^182^, ^188^ Specifically, the authors used this method to generate highly complex 3D scaffolds into which 3T3 fibroblast cells were encapsulated in a viable state for up to 5 d of culture (**Figure** **7**).^188^ In the authors' opinion, the combination of 3D printing and self‐healable hydrogel inks can pave the way for 3D‐printed tissue engineering constructs with the capacity to heal in the same way that natural tissues do within the human body. To this end, the authors also envision that the addition of shape memory properties to such constructs could enable the delivery of scaffolds with complex architectures using a noninvasive syringe injection methodology. We will revisit injectable scaffolds with shape memory and self‐healing capacity in Section 6.3.

#### 3.1.4 Gelatin

Gelatin is a natural protein that is derived from the denaturation of collagen via hydrolysis.^189^, ^190^ Consequently, gelatin retains the bioactive sequences of collagen (such as an arginine–glycine–aspartic acid peptide), while exhibiting limited antigenicity.^191^ Gelatin has numerous advantages making it an exciting biopolymer for tissue engineering and regenerative medicine.^148^, ^192^, ^193^, ^194^ This includes biocompatibility, biodegradability, cost effectiveness, and ease of modification.^190^, ^191^, ^192^ Yet, gelatin undergoes gel–sol transition at body temperature, which emphasizes the importance of chemical cross‐linking of gelatin‐based hydrogels for tissue engineering applications.^195^ Because gelatin contains aromatic residues in its backbone (e.g., tryptophan, phenylalanine, and tyrosine), the most logical method for developing self‐healable gelatin is through guest–host interactions.^196^ In a noteworthy example, Feng et al. used the guest‐host interactions between aromatic residues (guest) of gelatin and photo‐crosslinkable acrylate β‐cyclodextrin (Ac‐β‐CD) (host) to institute a highly stretchable (failure strain above 400%) hydrogel with quick healing ability (5 min) and good biocompatibility, as tested through encapsulation with human mesenchymal stem cells (hMSCs).^197^ The reversible nature of guest–host crosslinks made this hydrogel injectable and allowed infiltration and migration of hMSCs into the hydrogels without comprising the structural integrity of the hydrogel matrix. Most importantly, in vivo implantation of the hydrogel into rat calvarial bone defect led to enhanced tissue deposition in the defect, which was correlated with the ability of the hydrogel to recruit endogenous osteoblastic cells as the reversible crosslinks within the gel enabled cell migration without disrupting its mechanical integrity. As it was described in Section 2.4, double‐network hydrogels are another approach to produce self‐healing hydrogels. Along these line, gelatin methacrylate (GelMA) was used in conjugation with tannic acid (TA) to institute a self‐healing hydrogel with adhesive properties.^198^ The double‐network hydrogel in this study was comprised of precrosslinked GelMA hydrogel accompanied with TA as a multifunctional hydrogen bond provider. The resulting hydrogel showed significant increase in ultimate stress (4.3‐fold), compressive modulus (2.5‐fold), and elongation (sixfold), when compared to pristine GelMA hydrogel. Furthermore, the GelMA‐TA hydrogel generated sufficient adhesiveness to various surfaces (rubber, plastic, metal, glass, and porcine skin) corresponding with the TA content in the hydrogel.

### 3.2 Synthetic Polymers

Synthetic polymers have played an integral part in the technological revolution, which have been witnessed in modern times. This is in part due their chemical inertness, strong elastic modulus, flexibility, and ease of custom modification.^199^, ^200^ However, some synthetic polymers are highly toxic to humans, not biodegradable, and biologically inert, and thus not suitable for tissue engineering applications.^201^ Furthermore, many synthetic polymers are oil based, which is a great disadvantage, as the global oil supplies are expected to become greatly exhausted in the near future. Nevertheless, synthetic polymers offer a range of exciting properties that one has to consider when engineering self‐healing hydrogels. As was noted in the previous section, the natural‐based polymeric hydrogels are typically not stretchable, do not offer instant self‐healing, and are mechanically weak. Their synthetic counterparts can address all of these shortcomings, and we will, therefore, highlight their recent uses in the field of self‐healable tissue engineering hydrogels. In particular, we will mainly focus on self‐healable hydrogels made from polyethylene glycol, poly(acrylamide), and poly(vinyl alcohol) under conditions that make them amenable to tissue engineering applications (Figure 5).

#### 3.2.1 Polyethylene Glycol

**Figure 8:**
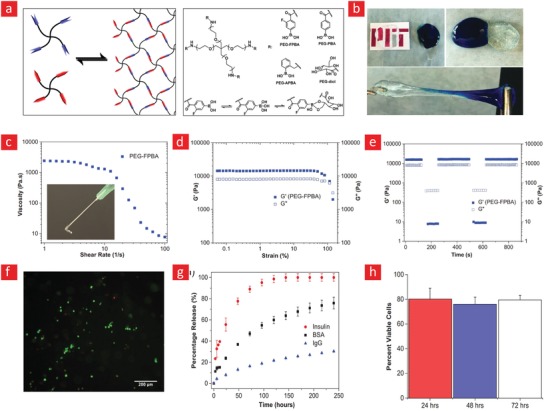
A stimuli‐responsive and self‐healable PEG‐hydrogel based on phenyl–boronic acid–cis–diol binding. a) The chemistry behind the self‐healing mechanism. b) Photographic images showing the self‐healing properties of the hydrogel. c) In addition to its self‐healing properties, this hydrogel was also shear‐thinning. d) Strain amplitude sweep and the associated e) step‐strain measurements of the PEG‐hydrogel to elucidate its self‐recovery properties. f) The viability of cells within the self‐healable hydrogels was ≈80% and remained at this level for up to 72 h. g) The release profiles of insulin, BSA, and IgG at different time‐points and h) cell viability at different time points are shown here. Adapted with permission.^122^ Copyright 2016, Wiley‐VCH.

PEG has been cemented as one of the most imperative hydrophilic polymers for biomedical applications thanks to desirable characteristics such as good biocompatibility, nonimmunogenicity, and versatile physical properties.^23^, ^202^, ^203^ The widespread recognition that PEG has garnered over the years has made it the first choice for most engineers attempting to develop self‐healing hydrogels. This is evident from the many studies published on the preparation of self‐healable PEG hydrogels.^57^, ^101^, ^122^, ^128^, ^204^, ^205^, ^206^ Examples include hydrogels with repair mechanisms based on borate esters,^122^, ^206^ Diels–Alder reactions,^128^ imine bonds,^205^, ^207^ hydrogen bonds,^57^, ^204^ and hydrophobic forces.^84^ Especially, a series of landmark achievements, wherein borate esters were used to impart good self‐healing efficiency and high mechanical stability are worthwhile mentioning.^122^, ^206^ In these milestone studies, the authors develop self‐healable hydrogels that were elastic and could reach high storage modulus values (up to 10 kPa) without rupturing. For instance, a team led by Robert Langer and Daniel G. Anderson managed by using boronate ester bonds between a four‐arm PEG–phenylboronic acid (three different PEG–phenylboronic acid derivatives: PEG–FPBA, PEG–PBA, and PEG–APBA) and four‐armed PEG–diol macromonomers, to develop a stretchable hydrogel with instant healing properties (**Figure** **8**).^122^ Moreover, this hydrogel displayed shear‐thinning properties and could thus be used as an injectable and self‐healable hydrogel carrier for stem cell therapy. However, to this end, it is pivotal that the hydrogel carrier can sustain viable cells over longer periods. The team validated this by encapsulating fibroblast cells within the hydrogel and demonstrated that the cells remained viable for up to 72 h with viability of around 80%. This team also examined the potential of this system for the targeted delivery of both biologics and cells to possible target sites in the body with promising results.

In another series of experiments, self‐healable PEG‐based hydrogels were synthesized by functionalizing telechelic difunctional PEG (DF‐PEG) with two aldehyde end groups through esterification of the hydroxyl groups on PEG and mixing the DF‐PEG with an aminated polymer such as chitosan^205^ or polyethylenimine (PEI).^207^ The combination of DF‐PEG and PEI resulted in an exciting hydrogel that could stretch up to 400% and recover fully to its original state. Moreover, once broken, it could instantly self‐heal and withstand extensive stretching without rupturing. However, one drawback of this study is the use of PEI, a molecule that can be toxic to cells at high doses.^208^ Along the same lines, a hydrogel comprised of dialdehyde‐functionalized polyethylene glycol (DF‐PEG) and agarose–ethylenediamine conjugate (AG–NH2) showed fast self‐healing (after 5 min) and exhibited higher yield stress (≈ 2000 Pa) when compared to pure agarose (126 Pa) or agarose–PEG hydrogels (315 Pa).^209^ This hydrogel demonstrated excellent adhesion to porcine skin and after being applied on the incision of the skin, it was able to withstand a bursting pressure that was even bigger than the arterial blood pressure (120 mmHg), indicating the capability of the hydrogels to assist in wound healing. Furthermore, human umbilical vein endothelial cells (HUVEC) in contact with the hydrogel extracts demonstrated high cellular viability (>80%), indicating low cytotoxicity of the hydrogels. Most importantly, hydrogels showed remarkable hemostatic capability after being applied to a rabbit liver incision immediately, in contrast with conventional sterile gauze.

Another way of preparing self‐healing PEG hydrogels is through thiol‐ene interactions, where the PEG alkene (or alkyne) reacts with a thiolated polymer to establish a self‐healing polymeric network.^210^, ^211^, ^212^ To this end, Macdougall et al. developed a self‐healing hydrogel from a mixture of four‐arm PEG alkyne and difunctional linear PEG thiol, yet this hydrogel suffered from low compressive strength and stretchability.^212^ In an attempt to address these shortcomings the same group developed interpenetrating networks (IPN) by incorporating a range of unfunctionalized natural polymers (alginate, chitosan, gelatin, heparin, or hyaluronic acid) into the hydrogel. Accordingly, PEG/alginate and PEG/gelatin systems showed higher compressive strength, tensile strength, and stretch‐ability when compared to control PEG hydrogel. The observed properties were attributed to existence of a secondary electrostatic loose network that complemented the efficient nucleophilic thiol‐ene cross‐linking chemistry. Most remarkably, human mesenchymal stem cells encapsulated in PEG/alginate hydrogels showed higher cell viability after 72 h (95%) when compared to that of PEG‐alone systems (77%), which implied PEG/Alginate provided a cytocompatible matrix that supports cell growth.

Recently, UPy has also been shown to be a promising tool for making self‐healable PEG hydrogels owing to hydrogen‐bond‐assisted dimerization of the UPy moieties.^57^ Notably, PEG oligomers were functionalized with UPy and immersed in a buffered solution, in which they began to solidify into stable hydrogels through hydrogen bonding mediated by UPy. As this system was injectable, it was used for further down‐stream studies to examine its ability to deliver bone morphogenetic protein 7 (BMP‐7) into kidney tissue via a minimally invasive injection method. Therefore, this UPy‐based approach could potentially lead to significant clinical break‐through discoveries in the foreseeable future.

#### 3.2.2 Poly(vinyl alcohol)

Another synthetic, biocompatible, non‐toxic and water‐soluble polymer—PVA—has also been employed in numerous biomedical applications ranging from contact lenses to scaffolds and various drug delivery platforms.^152^, ^213^, ^214^, ^215^ PVA is unique because of its many hydroxyl groups, which, together with its high water retention properties, makes it an ideal candidate for self‐healing hydrogels. Accordingly, over the years, several PVA‐based self‐healing systems have emerged, in which the healing mechanism was based on reversible hydrogen bonds.^63^, ^216^, ^217^, ^218^ In one such example, a double‐network hydrogel consisting of PVA and PEG was combined to yield an exceptionally tough hydrogel that could reach an ultimate tensile strength of almost 1.3 MPa while exhibiting shape memory and self‐healing properties.^219^ The secret behind the many amazing properties of this PVA–PEG system stemmed mainly from the presence of weak reversible hydrogen bonds between PVA polymers that yielded deformability, and strong chemical crosslinks between PEG polymers that could keep the system intact in high strain regimes and, thus, enable a return to its original shape. Even though a plethora of seal‐healable hydrogels have been presented to the field of biomaterial science, this combination of high material toughness and self‐healing ability is rather unusual, as high stiffness typically goes hand‐in‐hand with strong and stable intermolecular bonds, which conflict with the unstable and highly dynamic links behind self‐healable systems. Similarly, a hydrogel blend made from PVA, borax, and nanofibrillated cellulose resulted in a reinforced self‐healable hydrogel with improved mechanical properties as a result of reversible interchain hydrogen bonding between PVA and the nanocellulose, and strong boronate ester links mediated among the PVA polymers by borax.^220^ In a similar vein, a mixture of PVA and 2APBA yielded a hydrogel that exhibited self‐healing ability not only in PBS but also in a culture media (with or without the serum).^221^ Moreover, encapsulated fibroblast cells (CCL‐151) or breast cancer cells (MDA‐MB‐231) exhibited good viability over the course of 7 d, indicating cytocompatibility of the materials. More remarkably, the self‐healing ability of this hydrogel was successfully used to create a dynamic coculture system by separately encapsulating fibroblast cells and breast cancer cells in the hydrogel pieces and subsequently connecting them. In our opinion, this study holds much value as it can inspire researchers to create more complex culture environments for probing dynamic cell−cell and cell−matrix interactions.

Another challenge in the field of biomaterial science is the design and development of materials with high stiffness but fast molecular dynamics to yield a hydrogel that can quickly heal itself. To address this challenge, a recent study combined highly crystalline nanocellulose with PVA to generate a promising hydrogel for tissue engineering applications. Specifically, this self‐healable hydrogel was composed of hard, modified cellulose nanocrystals and soft, polymeric PVA domains crosslinked by dynamic host–guest interactions brought about by cucurbit[8]uril (CB[8]).^63^ This combination resulted in a mechanically strong hydrogel that could heal rapidly (within few seconds). From our viewpoint, such systems that address the conflict between strength and fast molecular dynamics could lead to new resilient biomaterials with the capacity to mend defects within load‐bearing tissues, such as cartilage and bone, as they can readily heal possible small fractures arising from the cyclic stretches and compressions imposed on natural bone and cartilage tissues.

#### 3.2.3 Poly(acrylic acid)/Poly(acryl amide)

Another class of synthetic self‐healing hydrogels—albeit less used—are those made from acrylate‐based polymers, especially acrylic acid and acrylamide. These hydrogels are special because of their good water absorption capacity and high density of functional chemical groups, such as carboxylic acid and amine groups, which in combination with their nontoxicity, makes them ideal to use for many biomedical applications.^222^, ^223^, ^224^, ^225^ Over the years, acrylate‐based self‐healable hydrogels have, for the most part been generated through hydrophobic interactions derived from either host‐gust interactions or micelles entrapped within the hydrogels.^81^, ^82^, ^226^, ^227^

In particular, the incorporation of micelles into poly(acrylic acid)/poly (acrylamide) hydrogels has proven a versatile pathway to produce self‐healable hydrogels with desirable attributes, such as high extensibility, elastic modulus, and toughness. A common approach to this end is the spontaneous polymerization of stearyl methacrylate (C18) into micelles within hydrophobically modified acrylic acid/acrylamide hydrogels in the presence of either of the surfactants; cetyltrimethylammonium bromide (CTAB) or sodium dodecyl sulfate (SDS).^81^, ^82^, ^226^ A key example of this methodology can be found in a recent study by Gulyuz et al.,^81^ wherein a poly(acrylic acid) hydrogel encapsulated with C18‐based micelles was developed. This self‐healable hydrogel could stretch up to 800% of its original length before it broke and displayed a high tensile strength in the range of 0.7–1.7 MPa. Similarly, the copolymerization of *N,N*‐dimethylacrylamide (DMA) and stearyl methacrylate (C18) in the company of SDS led to a poly(acrylamide)‐based hydrogel with self‐healing properties arising from the incorporation of C18/SDS‐based micelles within it.^82^ This hydrogel was extremely stretchable (broke at 4200% strain), displayed shape memory behavior, and could heal itself. However, one disadvantage of using the above‐mentioned methodology is the long hydrogel self‐healing duration; typically ranging from 20 to 60 min.

Another way of preparing self‐healing poly(acrylamide) hydrogels is through host‐guest interactions, in which the amine groups on the polymer backbone are functionalized with a host—cyclodextrin—and a hydrophobic and aliphatic guest molecule (*n*‐butyl acrylate, adamantane, and ferrocene).^61^, ^62^ Kakuta et al. embraced this approach to develop a self‐healable hydrogel based on a adamantine–ferrocene host molecule, which could immediately mend itself after rupturing, however, the self‐healing efficiency in terms of the adhesion strength between the rejoined pieces was relatively low, and it took up to 24 h to reach the prerupture adhesion strength.^61^ In another study, researchers attempted to develop a double‐network hydrogel by using host–guest interactions, between isocyanatoethyl acrylate modified with β‐cyclodextrin (host; β‐CD‐AOI_2_) and 2‐(2‐(2‐(2‐(adamantyl‐1‐oxy)ethoxy)ethoxy)ethoxy)ethanol acrylate (Guest; A‐TEG‐Ad), accompanied by a second covalent bonding between acrylate groups (achieved by UV‐initiated polymerization).^228^ The resulting hydrogel showed fatigue resistance and resistance to slicing because of the combination of host–guest interactions and covalent bonds in the hydrogel networks. Furthermore, in a strain sweep test (1% strain–1000% strain–1% strain), these hydrogels fully recovered their initial storage modulus even after the hydrogel had undergone several cycles. Most significantly, mouse bone marrow stromal cells (mBMSCs) or myeloid‐derived suppressor cells (MDSCs) cultured in the presence of the hydrogel extracts had a proliferation rate consistent with that of the positive control group, indicating their good cell compatibility. Most recently, the same group used a similar composition except they added GelMA to the recipe to benefit from its excellent biocompatibility.^229^ The resulting hydrogel exhibited superior mechanical properties compared to pristine GelMA and was able to completely heal itself after 1 h. Owing to shear‐thinning properties of this hydrogel, it was successfully 3D printed into a multilayer scaffold and mouse bone marrow stem cells (mBMSCs) cultured on top of these scaffolds were shown to be viable and proliferating after 7 d of culture. Furthermore, subcutaneous implantation of this hydrogel on the backs of nude mice (for 40 d) revealed that the scaffolds were completely integrated with the autogenous tissue of the nude mice, and new subcutaneous muscle tissues and blood vessels were formed in their pores with no immunological rejection occurred. These results suggested that these self‐healing scaffolds had favorable bioactivity and histocompatibility, making them useful in biomedical applications.

Hydrogen bonds are another alternative interaction scheme, which has been explored extensively over the years to develop self‐healable acrylic acid/acrylamide hydrogels.^53^, ^60^, ^230^ In one such study, acrylic acid and acrylamide were mixed in the company of glycogen to polymerize into a hydrogel through hydrogen bonds between carboxyl groups and hydroxyl groups in glycogen. This hydrogel could completely heal itself at neutral pH after 12 h while exhibiting shear modulus values as high as 1000 kPa and a swelling ratio that could reach 3500% after ≈ 500 min of swelling in water.^230^ The combination of self‐healing capacity at neutral pH together with high mechanical strength makes this system interesting, as many of the self‐healing hydrogels reconnect in nonphysiologically conditions and display low elastic modulus values. Based on the same concept, a double‐network hydrogel composed of poly(acrylamide‐*co*‐acrylic acid) (PAM‐*co*‐PAA) and PVA was generated via copolymerization and hydrogen bonds between the carboxyl groups of PVA and acrylamide groups of acrylamide. Moreover, the PVA polymeric backbone was made highly crystalline through a well‐established freeze/thawing procedure. Overall, this double‐network hydrogel could stretch up to almost 600% and reach 1230 kPa in tensile strength before breakage. The authors speculated that these formidable mechanical properties arose from the combination of weak reversible hydrogen bonds and the rigid crystalline PVA domains.^53^

### 3.3 Others

Other self‐healable hydrogels based on peptides,^231^, ^232^, ^233^, ^234^, ^235^ mussel‐inspired proteins,^47^, ^48^, ^101^, ^236^, ^237^ conductive polymers,^85^, ^233^, ^238^, ^239^, ^240^, ^241^, ^242^, ^243^, ^244^, ^245^ and zwitterionic polymers,^246^, ^247^, ^248^, ^249^ have also gained acceptance in the field. Peptide‐based hydrogels with self‐healing properties have been described elsewhere in detail, and the interested reader is referred to these recent reviews on this topic.^231^, ^250^ In this section, we will instead focus on conductive, mussel‐inspired and zwitterionic self‐healable hydrogels.

**Figure 9:**
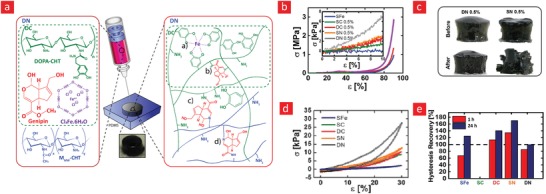
A tough, durable, and self‐healable chitosan–dopamine–hydrogel based on chelation. a) The chemistry behind the self‐healable hydrogel. DN indicates a double‐network hydrogel made from dopamine–chitosan (DOPA–CHT) and medium molecular weight chitosan (M*_M_*_w_‐CHT), while DC indicates a double crosslinked DOPA–CHT hydrogel made through genipin (crosslinks the amine group in CHT) and Fe^3^ (mediates coordination bonds between catechol groups). b) Compressive stress–strain curves of DOPA–CHT hydrogels that are solely crosslinked through Fe^3+^ (SFe) or through the addition of 0.5% genipin (SC 0.5%). SN (0.5%) and DN (0.5%) indicate single‐network DOPA‐CHT hydrogel with 0.5% genipin and double‐network hydrogel with 0.5% genipin, respectively. c) The self‐healing properties of DN 0.5% and SN 0.5% hydrogels are shown. d) Cyclic stress–strain curves and e) their associated hysteresis recovery. Adapted with permission.^102^ Copyright 2017, Wiley‐VCH.

The working mechanism behind most mussel‐inspired hydrogels stems from metal chelating cross‐links between positively charged Fe^+3^ ions and negatively charged catechol molecules.^48^, ^101^, ^102^, ^237^ In simple terms, the polymer in question is functionalized with negatively charged catechol groups—typically in the form of 3,4‐dihydroxyphenylalanine (DOPA)—which then crosslinks into a hydrogel through sacrificial bonds mediated by metal‐catechol coordination. These bonds are stronger than hydrogen bonds yet sufficiently weaker than covalent bonds to enable dynamic binding schemes and, ultimately, good self‐healing properties. A recent forerunner toward such Fe^+3^–DOPA‐mediated hydrogels was based on a DOPA‐chitosan (DOPA‐CHT) derivative, which could spontaneously crosslink into a double‐network hydrogel in the presence of Cl_3_Fe.6H_2_O (metal–ligand coordinate) and genipin (covalent) (**Figure** **9**).^102^ This bioinspired hydrogel displayed a range of interesting properties including: i) a high compressive strength in the MPa range, ii) good cytocompatibility and iii) fast self‐healing time ranging from 8 to 15 min. In addition, the mussel‐inspired hydrogel was injectable and could easily recover from repeated cyclic loadings. For these reasons, this is an ideal hydrogel carrier for stem cell therapies targeted against load‐bearing tissues, such as bone and cartilage.

Mussel‐inspired hydrogels with self‐healing capacity have also been generated under metal‐free conditions by using catecholamine‐3,4‐dihydroxyphenethylamine (dopamine) instead of DOPA. These hydrogels typically obtain their self‐healing properties from noncovalent interactions between aromatic rings, hydrogen bonds, and via imine bonds mediated by the NH2‐groups in polydopamine. An elegant solution toward such hydrogels was recently reported, wherein gelatin was functionalized with aldehyde groups and mixed with polydopamine under metal‐free condition (via sodium periodate) to generate an injectable, moldable, self‐healable hydrogel with fast recovery time, good self‐healing efficiency (95%), and a good adhesiveness (10–40 kPa).^237^ Such properties were an outcome of a complicated cross‐linking scheme comprised of hydrogen bonds in combination with imine and π–π interactions.

Another remarkable concept in the field is the development of electrically conductive and self‐healable hydrogels—as many tissues in the body—such as heart, muscle, and brain tissues are electroactive, and therefore need to be matched with similar electroactive biomaterials to yield good biointegration.^93^ Despite extensive studies on the design and development of self‐healable hydrogels, only a few studies have attempted to capture other properties, such as electrical conductivity. An elegant forerunner toward this goal has been based on polypyrrole‐based hydrogels; as polypyrrole is a highly conductive yet biocompatible polymer.^85^, ^233^, ^238^, ^239^, ^240^, ^241^

**Figure 10:**
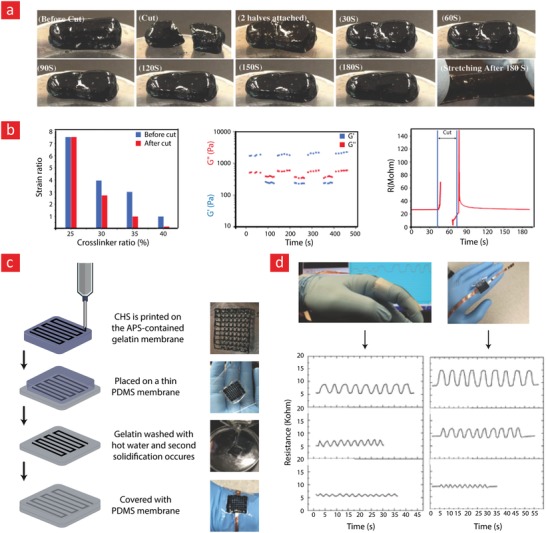
A conductive and self‐healable hydrogel made from chitosan (CSH), poly (acrylic acid) (PAA), and polypyrrole (PPY) based on electrostatic interactions. a) The self‐healing properties of the resulting hydrogel are displayed. b) The mechanical and electrical self‐healing efficiency of the hydrogel. c) Schematic depicting the preparation of the wearable sensor. d) The 3D printed wearable sensor could detect human‐body motions by measuring the associated resistance variation. Adapted with permission.^85^ Copyright 2017, Wiley‐VCH.

To this end, Darabi et al.^85^ generated a conductive, injectable, and self‐healing hydrogel by decorating chitosan with pyrrole and mixing it with acrylic acid monomers in the presence of Fe^+3^ and *N,N*˝‐methylenebis‐acrylamide (MBA) crosslinkers (**Figure** **10**). These components altogether generated a double‐network hydrogel compromised of reversible ionic interactions between carboxylic groups on poly(acrylic acid) and amine groups on polypyrrole mediated by Fe^+3^, and irreversible covalent bonds between neighboring poly(acrylic acid) chains mediated by MBA. The combination of reversible and irreversible bonds resulted in a highly conductive hydrogel that could stretch up to 1500% and which could self‐heal its electrical and mechanical properties after just 1–2 min. Specifically, the mechanical healing efficiency was complete (100%), whereas the electrical healing efficiency saturated at 96% after 1 min.

Another series of milestone concepts recently pursued is the development of electrically conductive, thermoplastic, moldable, and self‐healing hydrogels by mixing polypyrrole with agarose.^240^, ^251^ The polypyrrole makes the system conductive, while the agarose gel imposes self‐healing properties onto the system as the gelation of agarose is thermally reversible. This system could, therefore, heal itself through both external heat and near‐infrared light because of reversible liquidation and gelation in response to thermal stimuli. Moreover, the authors managed to adhere this conductive gel directly on the human skin for human motion detection and demonstrated that the system could yield an electrical circuit that could self‐heal via external heat or near‐infrared light stimuli.^240^, ^251^

Most recently, a new class of self‐healing hydrogels based on zwitterionic polymers have emerged— taking the field by storm owing to their unique biocompatible attributes. These polymers contain a balanced pairs of cationic and anionic groups, and mimic the phospholipids comprising the membranes of native cells or the mixed‐charge surfaces of many proteins.^246^ In fact, the positive and negative charges of the overall neutral zwitterionic molecules make a high dipole moment and such strong dipolarity endows excellent adhesion of zwitterionic hydrogels to many surfaces through ion–dipole or dipole–dipole interactions.^252^ Furthermore, the association of zwitterionic polymers can provide physical cross‐linking to enhance the mechanical properties of hydrogels. Most remarkably, zwitterions can assist the ion transportation along the highly dipolarized skeleton to promote ion conduction, which endows zwitterion hydrogels with good conductivity.^253^ Despite such great properties, most zwitterionic hydrogels are mechanically weak, hence researchers have incorporated other functionalities into them to make them self‐healable. In a noteworthy study, researchers utilized two different types of zwitterionic monomers, carboxybetaine acrylamide monomers with either one‐carbon (PCB‐1) or two‐carbon (PCB‐2) spacing between the charged groups, and separately cross‐linked them using carboxybetaine diacrylamide.^246^ Both hydrogels exhibited self‐healing behaviors in a strain sweep test (1% strain–300% strain‐1% strain), owing to ionic and hydrogen bonds between polymeric chains. Yet, PCB‐2 hydrogels demonstrated higher storage modulus compared to PCB‐1 hydrogels, which was attributed to stronger hydrogen bonds in PCB‐2 hydrogels. Additionally, both hydrogels were injectably and human embryonic kidney cells (HEK‐293T cells) encapsulated in these hydrogels retained a high cell viability even after being injected through a 28‐gauge needle. Additionally, hMSCs encapsulated in these hydrogels had greater population expansion over 14 d than that of cells in standard flask culture. Notably, hMSCs encapsulated in the hydrogels maintained their multipotency after 28 d of culture, while half the population of flask‐cultured hMSCs lost their multipotency. This phenomenon was shown to be a result of ROS‐scavenging capacity of zwitterionic hydrogels, which in turn causes stem cells favoring self‐renewal and mitigating nonspecific differentiation. In summary, this hydrogel presented a promising new platform for a wide variety of clinical applications requiring biocompatible injectable materials.

Another approach to make zwitterionic‐based self‐healing hydrogels is by modification of these polymers with boronic acid to allow formation of boron ester bonds. Along these lines, a group of researchers used a zwitterionic monomer (2‐methacryloyloxyethyl phosphorylcholine; MPC) and copolymerized it with a benzoxaborole‐containing monomer (5‐methacrylamido‐1,2‐benzoxaborole) to yield the PMB hydrogel. Separately, they have also copolymerized the MPC monomer with a glucose‐containing monomer (2‐gluconamidoethyl methacrylamide) to make the PMG hydrogel. Accordingly, mixture of PMB and PMG resulted in the formation of a hydrogel (PMBG) that showed fast self‐healing (after 20 s) owing to boron–ester bonds between the boronic acid (on PMB backbone) and hydroxyl groups (on PMG). The resulting PMBG hydrogels was injectable and showed pH‐responsive behavior due to the nature of boron–ester bonds. Additionally, both normal skin fibroblast cells (NSFB) and cancerous HeLa cells treated with the gel extracts maintained a high level of cell viability (>80%).^247^ Similarly, the same group copolymerized the MPC monomer with catechol‐containing monomer (dopamine methacrylamide; DMA) to yield poly (MPC‐*co*‐DMA) and, mixed it with poly (MPC‐*co*‐DMA) to facilitate a rapidly self‐healing hydrogel (after 1 min) with pH‐responsive behavior.^248^

### 3.4 Outlook and Future Opportunities

In summary, the synthetic polymers give rise to stronger self‐healable hydrogels with faster self‐healing kinetics than their natural counterparts. However, this is accompanied by much lower biocompatibility and potential adverse reactions within the body. The incorporation of cell adhesive motifs, such as RGD peptides, into the polymeric backbone of synthetic hydrogels, could potentially address some of these issues by enhancing the spreading, proliferation, and differentiation of encapsulated cells into mature tissues. The combination of a synthetic and natural polymer also offers the interesting possibility of generating a hydrogel that taps into the positive properties of both realms.

As the field advances, we anticipate that other sophisticated polymers grown inside bacteria—through recombinant technology—will grab the attention of scientists in the field, as this methodology can enable highly customized routes toward the generation of polymers with even better self‐healing efficiency and mechanical properties. Although attempts have already been made to introduce this technology into the field, most of these hydrogels have been weak (typically below 5 kPa) and, for the most part, intended to be used as injectable stem cell carriers that could mechanically shield the cells during the injection phase and retain them within the target site in a postinjection scenario.^231^ We, therefore, conjecture that further research into this area could yield some exciting self‐healable hydrogels for the field of tissue engineering.

## 4 Nanomaterial‐Based Self‐Healing Hydrogels

Nanomaterial‐based hydrogels are defined as hydrated polymeric networks held together by noncovalent or covalent bonding with each other and nanomaterial reinforcers.^93^, ^254^ These nanomaterials are incorporated into the hydrogels to either reinforce their physical properties (e.g., mechanical properties, thermal and electrical conductivity, and swelling degree) or to bestow superior biological properties to them.^255^, ^256^, ^257^, ^258^, ^259^, ^260^, ^261^, ^262^, ^263^ Accordingly, such nanocomposite hydrogels are highly desirable from a biomedical point‐of‐view, as many‐at‐the‐same‐time modifications are no longer needed to yield the combinatorial biomaterials required for optimal tissue regeneration.^90^, ^254^, ^259^, ^264^, ^265^, ^266^, ^267^ Recently, scientist have been working toward a new scenario, in which the nanomaterials are used to add autonomous repairing mechanisms to hydrogels.^268^, ^269^ Indeed, the combination of the multifunctional nature of nanomaterial‐based hydrogels with self‐healing characteristics would offer a promising prospect for generating even better tissue engineering hydrogels.^270^, ^271^, ^272^, ^273^, ^274^, ^275^, ^276^, ^277^ Overall, the nanomaterials can be categorized into three groups: a) carbon‐based, b) mineral‐based, and c) magnetic ones. The carbon‐based group typically refers to carbon nanotubes (CNT) and graphene,^278^ whereas the mineral based refer to clay‐based platelets and ceramic nanoparticles^254^ while the magnetic nanomaterials mostly include iron oxides.^279^ In this section, we will highlight some of the most important advances in generating such self‐healable hydrogels.

### 4.1 Carbon Based

Carbon‐based nanomaterials, such as CNT and graphene, represent a unique class of materials with fascinating properties, including excellent mechanical,^280^ electrical,^281^, ^282^, ^283^ and optical properties.^284^ They are currently widely used in the field for the design and development of tissue engineering scaffolds with the capacity to generate electroactive and load‐bearing tissues.^93^ Because carbon‐based nanomaterials are easy to modify they have also steered much attention among researchers as a new pathway toward self‐healable hydrogels; their popularity stems primarily from the many unique physical and chemical nanomaterial interactions, which they can establish with the polymer backbone of hydrogels. Through manipulation of these interactions, a range of self‐healable systems for tissue engineering applications has been developed in recent years, including hydrogels with hard‐to‐get properties, such as high self‐healing efficiency, good electrical conductivity, flexibility, and mechanical toughness.^274^, ^285^, ^286^, ^287^, ^288^ The following subsections will first describe the many chemical and physical properties of CNTs and graphene, and then the potential of these attributes in the engineering of self‐healable hydrogels with multifunctional capacities.

#### 4.1.1 Graphene

Graphene can be described as a sheet of 2D monolayers of sp^2^‐hybridized carbon atoms that are arranged into a high‐aspect ratio hexagonal pattern.^289^, ^290^ Since its discovery in 2004, some extraordinary properties have been associated with graphene. For instance, graphene is a potent conductor of electricity (2.50 × 10^5^ cm^2^ V^−1^ s^−1^) and heat (3000 W m^−1^ K^−1^)^291^ and has, in some cases, been reported to have a specific tensile strength 100 times that of steel.^292^ Another exciting property of graphene is its large surface area (2600 m^2^ g^−1^), which is an important contributing factor to the many strong interactions that it can establish with polymers. Graphene can be modified into an even more functional nanomaterial, graphene oxide (GO), which offers numerous modifiable oxygen‐based groups, such as OH‐groups, useful for further downstream applications.^293^ The electrical conductivity of graphene can also be further improved by reducing GO into reduced GO (rGO) through chemical and thermal treatment,^294^ however, this pathway can sometimes lower the quality of graphene and thus presents a tradeoff between chemical functionality and conductivity.^295^

The negatively charged OH‐groups on GO enable many interaction schemes with polymeric chains that can lead to self‐healable hydrogels. To date, most of these schemes have been based on either hydrogen bonds^286^, ^296^, ^297^ or chelating‐crosslinks mediated by metal ions.^54^, ^298^ The polymers used have for the most part been derived from acrylamide^47^, ^286^, ^296^, ^297^, ^299^ or acryl‐based polymers.^54^, ^298^ In general, the GO‐acrylamide hydrogels displayed much better properties than the acryl‐based ones, as they could elongate up to 4900%, displayed higher fracture strengths ranging from 0.18 to 1.0 MPa, and could heal more efficiently with a self‐healing efficiency that in some instances could reach up to 97.9% after 4 min of healing time.^297^ On the other hand, the GO‐acryl‐based hydrogels broke at strain values in the range 1000%–3000% with fracture strengths ranging from 0.07 to 0.8 MPa. GO‐acryl‐based hydrogels also displayed low healing efficiency, which at best could reach 50% after 48 h of healing time.^298^ Thus, it is clear that the GO‐acrylamide hydrogels are most worthy of attention and we will accordingly focus on them in this section.

An elegant forerunner to this end was a self‐healing GO‐acrylamide hydrogel that exhibited high elongations (4900%) with an ultimate tensile strength value of 0.35 MPa.^286^ Although this hydrogel displayed some amazing mechanical properties, its nonoptimal self‐healing efficiency (88% after 24 h) significantly limited its use in tissue engineering applications. This low self‐healing efficiency most likely stemmed from the incorporation of GO into the system as the healing efficiency dropped concomitantly with GO concentration. The authors speculated that this effect was caused by the large GO sheets acting as diffusion barriers, thus preventing the polymers from reconnecting broken cross‐links in the broken hydrogels. This lack of methodology was also evident in another publication by Han et al.,^47^ wherein the authors incorporated PDA into the GO‐acrylamide system. In brief, these authors used PDA to reduce GO to rGO, after which acrylamide monomers were added to the system in the presence of an initiator to commence the formation of a fully crosslinked hydrogel. The inclusion of PDA into the system had two functions: one that resulted in higher conductivity by turning GO into rGO and the other enabled the system to heal itself after damage. The healing capacity was caused by some reversible bonds between neighboring PDA chains, such as hydrogen bonding and cation–π interactions, as well as hydrogen bonds between PDA and polyacrylamide. In addition to all of these exciting properties, this hydrogel could also stretch up to 40 times its original length before breaking and exhibited an ultimate tensile strength that could reach almost 0.2 MPa under certain conditions. These additional mechanical properties were achieved through the combination of the above‐mentioned reversible bonds and strong covalent bonds between the polyacrylamide chains. Although the self‐healing efficiency was less than optimal (80% after 24 h), the system provided some exciting opportunities, such as reasonable adhesive strength to skin tissue (30 kPa), long‐term biocompatibility in vivo, high conductivity, and a good electrical healing efficiency (95% after 24 h). Overall, these exciting properties made the system able to perform various tasks, such as measuring the cyclic movement of articular joints without failing, detect electromyographic signals from the skin, and regenerate broken cartilage tissue in a rabbit animal model.

Some studies have also focused on the incorporation of graphene into hydrogels that were made from much smaller polymers such as amino acids,^300^ amine‐terminated branched oligomers,^136^ and DNA strings.^301^ The most noteworthy of these are, in the author's opinion the one based on branched oligomers. This hydrogel could self‐assembly spontaneously after mixing carbonyl‐functionalized GO sheets with the branched and amino‐rich oligomers through imine bonds mediated between the GO sheets and the NH2‐groups, as well as hydrogen bonds between neighboring NH2‐groups. In its essence, the developed system was, therefore, a double‐network hydrogel, and the end‐product was a strong mechanical hydrogel with fast self‐healing time. Specifically, the GO‐based hydrogel could reach a tensile modulus close to 1 MPa while offering self‐healing properties. These healing properties included a self‐healing efficiency close to 100% after 1 h of healing at room temperature. However, just as in some of the other studies reviewed herein,^47^, ^286^ the self‐healing efficiency dropped significantly as function of graphene concentration. The authors speculated that this was caused by the many strong imine bonds established between GO and the polymer due to the large GO surface area, which in turn resulted in a higher density of bonds that were dynamic, but yet, much stronger and stable than hydrogen bonds. The authors propose that these issues severely restricted the movement of polymer chains; this was also reflected in their differential scanning calorimetry (DSC) results, as the glass transition temperature (*T*_g_) increased significantly from −5 °C to 9 °C with increasing GO concentration (1% → 4%).

In conclusion, GO‐based self‐healable hydrogels have added exciting attributes such as good conductivity, sensing‐abilities, and increased mechanical strength to self‐healable hydrogels. For these reasons, there is no doubt that the inclusion of GO in the field has led to some interesting scientific possibilities. However, these additional opportunities have come at the cost of a much lower self‐healing efficiency caused by restricted polymer mobility mediated by the large GO sheets. Therefore, further investigations are needed to develop new systems that can address this lack‐of‐methodology and unleash the full potential of GO‐based self‐healable hydrogels.

#### 4.1.2 Carbon Nanotubes

Since their discovery in 1991,^302^ CNTs have been applied widely in the field of materials science, largely due to their electrical and mechanical properties.^303^ The unique electromechanical properties of CNTs have made them useful in a great variety of applications, such as in electronics, optics, biomedical engineering, and nanotechnology.^304^, ^305^, ^306^ The huge interest in CNTs is evident from the annual production of CNTs, which is currently in the megaton range and rapidly increasing. Indeed, CNTs are already being used in some commercial products, including water filters, rechargeable batteries, automotive parts, and sporting hulls.^307^

CNTs can be described as sheets of carbon atoms that are rolled into high‐aspect‐ratio (>1000) nanotubes. These carbon atoms are arranged in well‐ordered hexagonal structures mediated by sp^2^ bonds between neighboring carbon atoms. The CNTs themselves can have diameters ranging from 0.8 to 20 nm—depending on whether they are single or multiwalled—with lengths ranging from a couple of nanometers to several centimeters.^307^ Additionally, CNTs can be functionalized with highly reactive groups, such as hydroxyl, carbonyl, carboxyl, and amines and are, therefore, easy to incorporate into various hydrogel systems. They also pose unique properties such as high tensile strength (11–63 GPa),^308^ electric conductivity (10^9^ A cm^−2^),^309^ Young's modulus (1–1.8 TPa),^295^ thermal conductivity (2000–6000 W m^−1^ K^−1^ at room temperature),^310^ and a specific tensile strength which outcompetes that of steel by up to 100 times.^311^ CNTs are, therefore, perfect reinforcing agents for self‐healable hydrogels as they are mechanically strong, easy to functionalize, thermally conductive, and electrically active.

Compared to the many exciting reports on GO‐based self‐healable hydrogels, the literature on the CNT‐based hydrogels is rather sparse.^287^, ^312^, ^313^, ^314^ Indeed, in our opinion, these systems need much future attention to fully open up the field of multifunctional and self‐healable hydrogels. Nevertheless, two recent studies from 2014^287^ and 2017^314^ have led to a series of milestone contributions in this regard. In one of these studies, a supramolecular hydrogel based on both weak and strong hydrogen bonds was made from carboxylic functionalized CNTs and polyethylene polyamine (PPA). In brief, this system consisted of weak intermolecular hydrogen bonds (N—H···N) between individual PPA molecules and strong bonds (N—H···O) between PPA and the carboxylic groups on CNTs. This hierarchical bonding scheme gave the system self‐healing, adhesive, and sol–gel properties. Specifically, the system could restore 90% of its mechanical properties within 90 s and adhered to a Teflon surface with an adhesion force of 10 kPa. The hydrogel also spontaneously solidified into a gel at 55 °C after 30 s and could return to its original liquid state when the temperature was shifted back to room temperature. Since CNTs are famed for their incredible thermal conductivity, this reversible sol‐gel transition could easily be controlled through external NIR light stimuli, and therefore stimuli responsiveness can be added to these systems already impressive list of properties.

**Figure 11:**
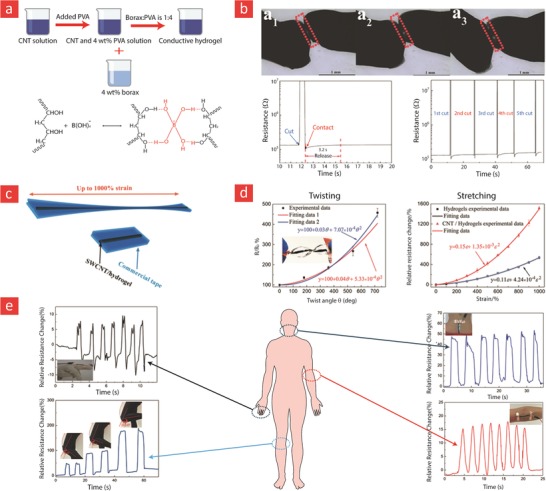
A conductive and self‐healable hydrogel made from CNTs and PVA, in which the self‐healing mechanism is governed by hydrogen bonds. a) The chemistry behind the self‐healable hydrogel. b) Photographic images of the self‐healing properties of the hydrogel and its electrical self‐healing efficiency. c) The hydrogel was embedded within a VHS scotch tape to increase its d) twistability and stretchability (up to 1000%). e) The hydrogel was utilized in a sensing device for the detection of various human motions. Adapted with permission.^314^ Copyright 2016, Wiley‐VCH.

In another study, a self‐healing, conductive hydrogel was created through the incorporation of CNTs and borax into a PVA hydrogel (**Figure** **11**).^314^ The self‐healing mechanism in this hydrogel was also primarily based on reversible hydrogen bonds, which in this case was generated between PVA and borate ions. Interestingly, the device was embedded into a Scotch permanent clear mounting tape to enable it to stretch up to 1000%. Specifically, the device could heal after 3 s with a self‐healing efficiency of 98%; and because of the CNT reinforcement, this hydrogel system was also tough, highly conductive, and piezoelectric. The piezoelectric properties of the system were used to turn the device into a high‐fidelity sensing device. To this end, the authors demonstrated a remarkable human‐motion sensing capacity of the device by utilizing its piezoelectric properties to sense resistivity changes during human‐joint motions.

In conclusion, despite their great promise as self‐healing and multifunctional reinforcing agents for tissue engineering hydrogels, CNTs have not yet been fully used to generate such advanced hydrogels. However, a few exciting studies have emerged, which have demonstrated the huge hidden potential of CNT‐based self‐healable hydrogels. Indeed, we expect interesting developments in CNT‐based hydrogels in the coming years.

### 4.2 Mineral Based

Minerals such as calcium, silicate, lithium, magnesium, phosphate, and zinc are important components in bone and cartilage. In fact, it is the mineral phase of bone and cartilage that gives them their incredible load‐bearing properties, which in turn enables the human skeletal system as a whole to withstand repeated mechanical stimuli directed at the body during daily routine activities.^315^, ^316^, ^317^ For these reasons, mineral‐based nanomaterials made from clay materials (e.g., montmorillonite and laponite), bioactive glasses, calcium phosphates, and calcium carbonates have been used extensively in recent years to make hydrogels more appealing for skeletal tissue engineering, providing a manifold increase in load‐bearing and osteogenic properties compared to pristine hydrogels.^93^ Nevertheless, most of these hydrogels cannot resist the cyclic in vivo biological forces in skeletal tissues for long periods; instead, they quickly rupture and disperse into the body. Therefore, the design and development of mineral‐based hydrogels that can self‐heal within the load‐bearing microenvironment of skeletal tissues are needed to transform these systems into truly “master‐healers” of damaged skeletal tissues. In this section, we will review various self‐healable systems with a specific focus directed towards clay‐based nanoreinforcments, as they, in our opinion, constitute the key to overcoming the current challenges in bone tissue engineering.

#### 4.2.1 Nanoclays

Silicate is the principal component of nanoclays, but nanoclays are much more than just nanosized silicate, as they can contain traces of important skeletal minerals such as magnesium, calcium, zinc, and lithium.^93^ Nanoclays are also potent mechanical nanoreinforcers by virtue of their ultrathin (≈1 nm) and high‐aspect ratio geometry (up to ≈1000), and like their carbon‐based counterparts, they also display a high‐surface area consisting of many easy‐to‐modify OH‐groups, which enables a multitude of possible self‐healable crosslinks with the polymeric backbone of hydrogels. These properties make nanoclays ideal for use as multifunctional and self‐healable agents for skeletal tissue engineering. In recent years, several studies have demonstrated that the above‐mentioned feats of nanoclays are realistic in real‐world applications.^93^, ^254^, ^318^ Most of these studies have been centered on either laponite or montmorillonite; two of the most well‐renowned clays in this field. Therefore, in this section, we will focus on laponite‐ and montmorillonite‐based self‐healable hydrogel systems.

**Figure 12:**
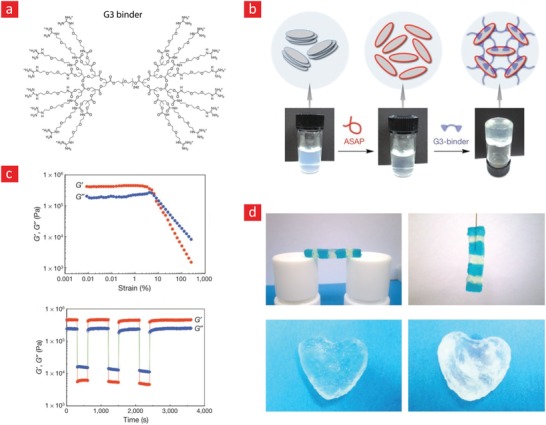
An injectable and nanoreinforced hydrogel with self‐healing properties based on electrostatic interactions between laponite and a G3 binder. a) The chemistry behind the self‐healable hydrogel. b) Photographic images of the hydrogel crosslinking process. c) The shear‐thinning and self‐healing properties of the resulting hydrogel were examined through rheology. d) Photographic images showing the self‐healing and shape memory properties of the laponite‐based hydrogel. Reproduced with permission.^64^ Copyright 2012, Macmillan Publishers Ltd.

Laponite is perhaps one of the most widely used nanoclays in skeletal tissue engineering since several studies have demonstrated its ability to turn bone‐marrow‐derived stem cells into bone cells.^8^, ^93^, ^259^, ^319^ In brief, laponite is a silicate‐based nanoplatelet with a diameter of 25 nm and a thickness of 1 nm.^93^ It contains traces of lithium, magnesium and natrium and displays a negative charge, which makes it easy to disperse in water at low concentrations. In general, the working principle behind laponite‐based self‐healable hydrogel is a result of ionic interactions between negatively (at the face) or positively (at the rim) charged sections of laponite with charged sections present within the backbones of various polymeric systems.^64^, ^320^, ^321^, ^322^, ^323^, ^324^, ^325^, ^326^, ^327^, ^328^ In two groundbreaking studies, dendritic molecules consisting of amine‐terminated end groups (G3‐binder) were mixed with laponite to yield a moldable, freestanding, and self‐healable hydrogel; possibly because of the reversible ionic interactions between the negatively charged laponite and the positively charge amine groups on the dendritic molecules (**Figure** **12**).^64^, ^325^ This hydrogel was injectable, could heal in less than 1 h with a mechanical recovery close to 100%, retain almost 98% water, and was able to memorize its original shape after a drying and rehydration process. This combination of injectability and shape memory properties makes it well‐suited for various injectable tissue engineering strategies. In another study, laponite was mixed with a carboxybetaine methacrylamide (CBMAA‐3)–2,hydroxyethyl methacrylate (HEMA) polymer to yield a highly hydrated hydrogel, which could extend to 1800% of its original length before breaking, and rapidly heal fractures (within 5 min).^321^ A major advantage of these laponite‐based self‐healable systems is their simple working principle combined with their facile manufacturing process. This important attribute does not only make them readily injectable into the body, but also makes it much easier to bring them beyond the FDA approval phase and into the clinic.

Compared to laponite, montmorillonite is a less‐studied nanoclay in the field of skeletal tissue engineering, which makes the incorporation of montmorillonite into existing skeletal regenerative strategies an approach that is ripe for investigations. In the following paragraphs, we will highlight some of these promising systems with a special emphasis directed towards their self‐healing capacity. In simple terms, montmorillonite can be described as a negatively charge silicate clay with a thickness of 1 nm and a length that can range from 100–1000 nm.^93^ This makes montmorillonite a much higher‐aspect ratio nanomaterial than laponite and thus a better mechanical reinforcer. Similar to laponite, montmorillonite also contains traces of magnesium and natrium; however, unlike lithium, montmorillonite also contains significant amounts of aluminum.

The self‐healing mechanism in most of the reported montmorillonite‐based systems (like the laponite‐based systems) is governed by interactions between positively charged NH2‐groups on the polymer backbone within the hydrogel matrix and the many OH‐groups present on montmorillonite.^78^, ^329^, ^330^, ^331^ Gao et al. recently published an elegant forerunner to montmorillonite‐based systems by incorporating montmorillonite into a covalently crosslinked polyacrylamide hydrogel. In this study, the NH2‐groups present on polyacrylamide were able to physically interact with montmorillonite because of a combination of hydrogen bonds and possible interactions between the oppositely charged NH2 and OH‐groups, to yield a double‐network hydrogel. Indeed, because of its double‐bonded crosslinking nature, this hydrogel could stretch to 11800% its original length before breaking, and could easily twist into various complex shapes. Nevertheless, the self‐healing efficiency of this hydrogel was relatively poor; it took almost 3 d for the system to self‐heal. In another recent study,^331^ a similar approach was used to develop a system with a much better self‐healing property. In brief, the authors in this study choose to use a much smaller aminated polymer than polyacrylamide, namely a poly(amidoamine) dendrimer, which, because of its low molecular weight and higher mobility, was able to reconnect broken links much faster. Specifically, it was found that it only took 400 s for a broken hydrogel to reach its original shear modulus.

In conclusion, despite their great promise as self‐healing nanomaterials for tissue engineering hydrogels, the nanoclay‐based hydrogels have not yet reached a mature stage. Especially, the montmorillonite‐based systems, despite their amazing stretching properties, require further improvement to yield a self‐healing efficiency that matches their carbon‐based counterparts. In the author's opinion, this is for the most part linked to the large size of montmorillonite, which significantly increases the diffusion barrier within the hydrogel matrix and, therefore, puts severe restraints on the polymer mobility. However, the studies highlighted in this section have shown sufficient promise, which once fully addressed could result in exiting self‐healable hydrogels for skeletal tissue engineering.

#### 4.2.2 Others

Other self‐healable hydrogels embedded with mineral‐based nanomaterials, such as silicate,^332^, ^333^, ^334^, ^335^ hydroxyapatite,^273^ magnesium silicate,^271^ and calcium carbonate^86^ nanoparticles, have also been developed, with promising results. Of these, self‐healable systems made from silicate and calcium carbonate nanoparticles have shown the most promise.

**Figure 13:**
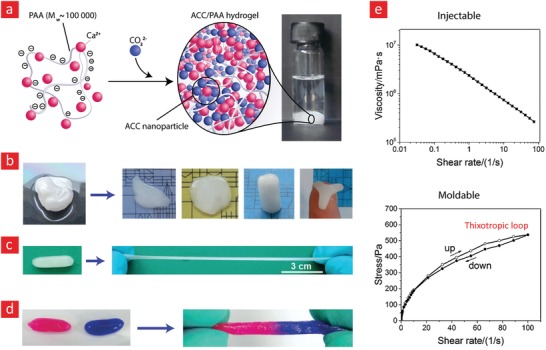
A self‐healable polyacrylic acid (PAA) hydrogel based on electrostatic interactions between PAA and amorphous calcium carbonate (ACC) nanoparticles. a) The chemistry behind the self‐healable hydrogel. Photographic images demonstrating the b) moldability, c) stretchability, and d) self‐healing properties of the hydrogel. e) The shear‐thinning and moldability of the hydrogel were measured through rheology and are displayed here. Adapted with permission.^86^ Copyright 2016, Wiley‐VCH.

The skeletal system of the human body utilizes some minerals to give it the load‐bearing support it needs during various day‐to‐day activities. For instance, minerals such as calcium carbonate and calcium phosphate have a role in the mechanical properties of bone, adding strength to and hardening the soft phase. In a recent study by Sun et al.^86^ calcium carbonate nanoparticles were implemented within a polyacrylic acid hydrogel to yield a biomineral‐like material with self‐healing capacity (**Figure** **13**). This system was made through a simple mixing of CaCl_2_, Na_2_CO_3,_ and polyacrylic acid in water followed by vigorous stirring. Scanning electron microscopy of the freeze‐dried hydrogel clearly showed the presence of nanosized calcium carbonate nanoparticles that were crosslinked with the hydrogel backbone. The resulting hydrogel exhibited shape memory properties, was moldable, injectable stretchable and could self‐heal within 5 s. The authors speculated that the driving mechanism behind the listed properties was reversible ionic interactions between Ca^2+^ and the negatively charged polyacrylic acid backbone. Especially, the combination of injectability, shape memory property, and rapid self‐healing makes this biomineral‐like hydrogel promising for bone tissue engineering applications, as it can readily be injected into the target size to form a stable and predetermined mineral structure.

Behind nanoclays, silicate nanoparticles are the second most frequently incorporated mineral‐based nanomaterial within self‐healable hydrogels.^332^, ^333^, ^335^, ^336^ Like the nanoclays, silicate nanoparticles also offer many modifiable OH‐groups, and therefore enable a multitude of self‐healing schemes. For example, in a recent study a polymer consisting of polyacrylamide and small traces of stearyl methacrylate was covalently grafted to silica nanoparticles to form a self‐healable hydrogel that could retain up to 90% water.^333^ In addition to strong covalent bonds, this system also consisted of hydrogen bonds mediated by the NH2‐groups of polyacrylamide and hydrophobic interactions among the stearyl methacrylate groups. The system was thus essentially a triple‐bonded hydrogel, which was a contributing factor to its mechanical properties. This hydrogel could stretch to 2830% of its original length before breaking and displayed an ultimate tensile strength of 256 kPa. Moreover, the hydrogel was able to heal itself with an efficiency of almost 70% after it was left broken at 60 °C for 24 h. The inclusion of silicate nanoparticles was an essential factor in reaching the above‐mentioned mechanical and self‐healing properties, and the authors speculated that this was caused by the ability of the silicate nanoparticles to delay the onset of crack propagations within its matrix by redistributing the applied mechanical stress.

In another study, positively charged poly(2‐dimethylaminoethyl methacrylate) was grafted onto silicate nanoparticles and then mixed with the highly anionic poly(acrylic acid) to yield a hydrogel that was crosslinked through electrostatic interactions between its oppositely charged polymers.^334^ Notably, the silicate nanoparticles enabled this hydrogel to effectively dissipate accumulated energy during the extension phase of the hydrogel, enabling it to reach 2000% strains before breaking. Moreover, due to the many reversible electrostatic interactions, this system could self‐heal with an efficiency ranging from 80% to 100% after 12 h of healing time. Besides its excellent stretchability and reasonable healing‐efficiency, the as‐prepared hydrogels also displayed shape memory behavior and were therefore highly suited as a hydrogel carrier for stem cells targeted against skeletal tissue disorders.

### 4.3 Magnetic Nanomaterials

In recent years, magnetic nanomaterials have been incorporated into tissue engineering hydrogels to make them responsive to magnetic fields and thus easier to control from outside the body using externally applied electromagnetic fields.^337^, ^338^, ^339^ This marriage between magnetic nanomaterials and tissue engineering hydrogels can, in the long run, yield stimuli‐responsive hydrogels that can actuate and heal on command within the human body. The working principle behind these systems relies on reversible links between the magnetic nanomaterial and the hydrogel backbone. However, such intelligent tissue engineering systems are still in their infancy, with only a few published studies. Most of these studies have focused on the incorporation of iron nanoparticles into hydrogels and have utilized catechol–iron coordination bonds to endow self‐healing properties to these systems. For instance, a mixture of magnetic iron nanoparticles and a four‐arm terminated polyethylene glycol (4cPEG) polymer enabled spontaneous hydrogel cross‐linking through metal coordination interactions between the polymer chains and iron nanoparticles.^338^ This hydrogel was magnetic, self‐healable, biocompatible, and stretchable (up to 1000% strain values). Despite the many interesting properties of the iron‐4cPEG system, its magnetic properties were, in the author's opinion, not fully utilized. It would be interesting to examine the self‐healing time and its efficiency as a function of an externally applied magnetic field.

Another hydrogel with both self‐healing and magnetic properties was also recently manufactured from the combination of chitosan and negatively charged iron‐coated graphene oxide (FeGO) nanomaterials.^339^ These films demonstrated self‐healing ability due to electrostatic interactions between positive amine groups on chitosan and the negatively charged FeGO nanomaterials. Specifically, the incorporation of FeGO into chitosan had a direct effect on the hydrophobicity of the resulting hydrogel, as well as its mechanical and magnetic properties. Interestingly, the FeGo–chitosan system also displayed some noteworthy antibacterial properties, which could be utilized to develop hydrogels that can regenerate chronic wounds, while at the same time reducing the probability of wound infection and possible foreign body responses. However, in our opinion, the authors of this study did not fully tap into the exciting magnetic properties of their system. Indeed, the area of magnetic and self‐healable tissue engineering hydrogels presents a host of yet unexplored scientific possibilities, which once fully harnessed might push the field of tissue engineering to new exciting highs.

### 4.4 Outlook and Future Opportunities

Attributes such as high mechanical strength, conductivity, and osteoconductivity have made nanomaterials suitable for various tissue engineering applications. In recent years, they have also been incorporated into soft matrixes to yield multifunctional hydrogels with self‐repair capacity. Even though some of these systems could deliver the promise of hydrogels that are both multifunctional and able to rapidly self‐heal, some of the nanomaterials, such as montmorillonite and graphene, significantly reduced the self‐healing efficiency to the point that made them almost useless in the clinic. We noted that this was most likely caused by their high‐aspect ratio, which in turn resulted in the formation of nanomaterial‐based barriers that prevented efficient polymer diffusion. One possibility to address this problem is to incorporate the nanomaterials into the hydrogel matrix in a more orderly fashion. For instance, the use of a honeycomb‐like nanomaterial network within the matrix—instead of a random network—could result in a significant mechanical enhancement, together with improved mass transfer through the many pores within the honeycomb network. In fact, some recent studies have shown that this is easy to achieve, as such networks can be generated within hydrogels by simply freeze‐drying the nanomaterials,^340^, ^341^ and then immersing them into a precursor hydrogel solution that is subsequently crosslinked into a solid construct. We also notice that the combination of a magnetic field and carbon nanotubes could yield long carbon nanotube treads^342^ that could easily be incorporated within various self‐healable hydrogels to exhibit truly outstanding tissue engineering materials both from a mechanical and electrical point‐of‐view.

However, despite the great promise of nanoreinforced and self‐healable hydrogels, their performance inside the body is still not fully elucidated. The major concern here is related to possible cytotoxic and immune responses in a postimplantation scenario from the carbon‐based nanomaterials, as they are nondegradable (in contrast to the mineral‐based nanomaterials), and therefore might elicit unwanted in vivo responses.^93^ Nevertheless, there are several roadmaps that could potentially reduce such outcomes by making the carbon‐based nanomaterials easier to degrade by the human body through various chemical functionalization strategies.^93^ As the field advances, we anticipate that this current gap between the laboratory and the clinic will be further minimized to enable the patients to fully benefit from the combined healing powers of self‐healable hydrogels and nanomaterials.

## 5 Tissue Engineering Applications

Besides, biological and structural factors, the native microenvironment of tissues within the body relies on mechanical (i.e., bone, muscle) and electrical cues (i.e., cardiac, nerve). Therefore, tissue engineering hydrogels, aiming to integrate with native organs also need to be durable from a mechanical point‐of‐view and (in some situations) also electroactive. In this regard, major efforts have been directed toward engineering of hydrogels that are simultaneously mechanically though, self‐healable and eletroactive. In this section, we will review some of the recent progress in these areas with special emphasis on musculoskeletal, cardiac, and neural tissue engineering.

### 5.1 Bone

Bone is the second most transplanted tissue in the world, which underscores the immense need for off‐the‐shelf bone grafts.^343^ Although the transplantation of fresh autologous bone is the current gold standard in the clinic, this option is limited due to the significant donor site morbidity associated with removal and reinsertion of the patients own bone tissue inside the target defect site.^344^ While allografts offer some exciting advantages, they are also associated with significant flaws related to possible foreign body responses and graft rejection.^154^, ^343^, ^345^ Consequently, bone tissue engineering has emerged as a promising alternative to auto and allografts, wherein stem cells, scaffolds, and biological factors are harnessed to create a native‐like microenvironment capable of facilitating the formation of new bone tissue.^346^, ^347^

One of the key requirements of bone tissue engineering is the design and development of tissue engineering scaffolds that recapitulate important characteristics of bone, including its mechanical strength, durability, and self‐healing capacity.^348^, ^349^, ^350^ Correspondingly, recent efforts have been redirected toward engineering of self‐healable scaffolds that can cope with the load‐bearing conditions in the native bone, while facilitating new tissue ingrowth. Thus far, a number of studies have explored the enormous potential of self‐healable hydrogels in this field, utilizing prepolymers such as HA,^182^, ^351^, ^352^ (PEG),^272^, ^353^, ^354^ elastin‐like polypeptides (ELP),^355^ chondroitin sulfate,^356^ protein–DNA complexes,^357^ and silk fibroin.^358^ These hydrogels have mostly relied on crosslinks based on thiol‐ene click chemistry,^353^ supramolecular interactions (involving DNA blocks),^357^ electrical interactions,^351^, ^352^, ^358^ guest–host interactions,^182^, ^197^ imine bonds,^355^ acylhydrazone bonds, and Diels–Alder reactions.^356^

**Figure 14:**
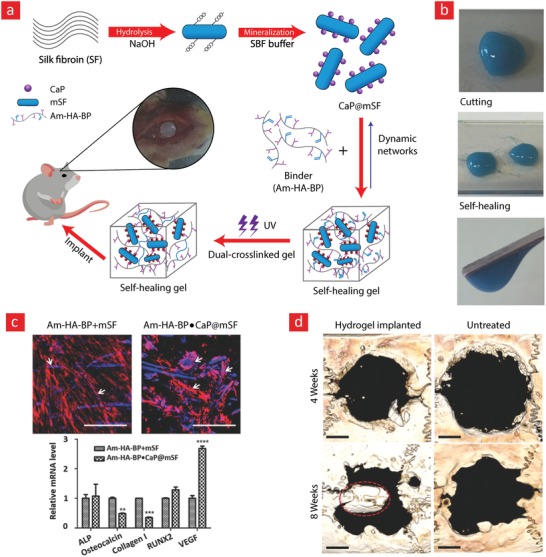
A silk‐based self‐healable hydrogel for bone tissue engineering. a) The chemistry behind the self‐healable hydrogel. b) Photographic images showing the self‐healing properties of the hydrogel. c) The hMSCs spreading within the hydrogels was examined through phalloidin (red) and DAPI (Blue) staining of the cell cytoskeleton and nucleus, respectively. The relative gene‐expression of important bone markers from hMSCs encapsulated within hydrogels is also displayed in this panel. d) The self‐healable hydrogel could promote significant bone formation within rat cranial defect site. Adapted with permission.^358^ Copyright 2017, Wiley‐VCH.

**Figure 15:**
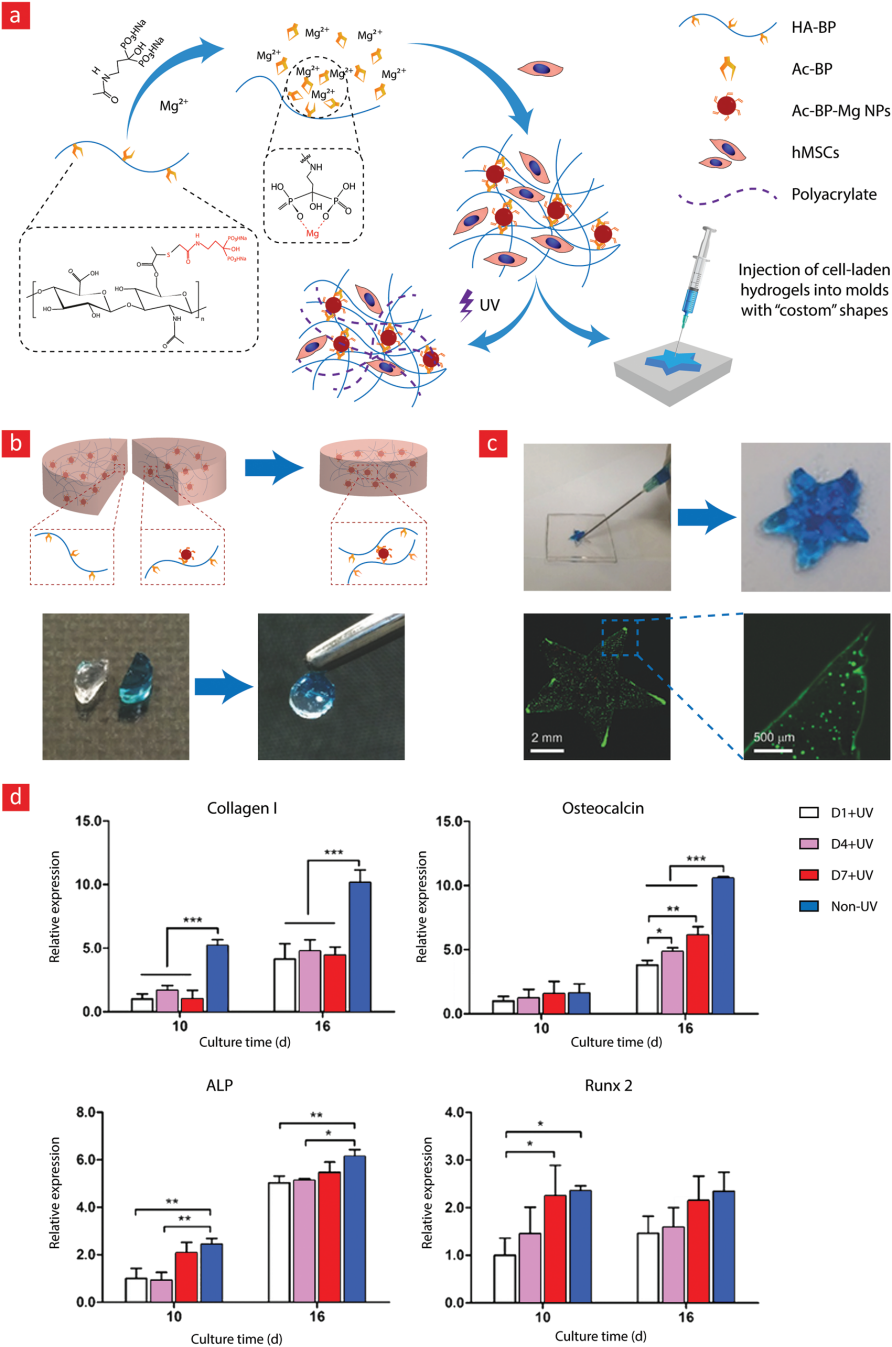
An HA‐based self‐healable hydrogel for bone tissue engineering. a) The chemistry behind the self‐healable hydrogel and its b) self‐healing and c) modability properties. d) The relative gene‐expression of important bone markers from hMSCs encapsulated within hydrogels is also displayed in this panel. Adapted with permission.^352^ Copyright 2017, Wiley‐VCH.

The most frequently used mechanism to form self‐healable hydrogels for bone tissue engineering is based on electrostatic attractions; wherein various ions are interacting with oppositely charged ligands on polymeric chains.^351^, ^352^, ^358^ For instance, in a recent study, HA macromolecules modified with bisphosphonate (BP) groups were shown to be able to bind reversibly to Ca^2+^ ions that were conjugated onto silk microfibers (mSF) through a calcium phosphate (CaP) coating (**Figure** **14**).^358^ Due to the presence of reversible electrostatic interactions, this mixture resulted in an injectable and self‐healable hydrogel. However, the silk‐based hydrogel demonstrated poor mechanical properties and insufficient stability in physiological condition as it quickly degraded (5 h) in PBS. To circumvent this drawback, the authors transformed this system into a double‐bonded one by adding a UV cross‐linkable HA–BP–acrylamide (Am–HA–BP) prepolymer to the system. This double‐network hydrogel showed significantly higher stability and an increase of ≈1500% in storage modulus. Moreover, the in vitro viability of encapsulated hMSCs was high, while the hydrogel itself showed some exciting osteogenic properties. Most importantly, results from an in vivo study in mouse revealed a significant acceleration in bone regeneration rate in a cranial size defect model, as the bone formation rate was 220% faster than in untreated groups.^358^ In another paramount study, electrostatic interactions were used to generate an injectable and self‐healable hydrogel by mixing HA‐BP and acrylated BP (Ac‐BP) with magnesium chloride (MgCl_2_) (**Figure** **15**).^352^ Interestingly, the shear thinning property, compressibility, and stress relaxation profile of this hydrogel allowed it to be injected and rapidly fit within custom‐made defect sites. Finally, encapsulation studies with hMSCs in these nanocomposite hydrogels demonstrated their capacity to induce cell spreading and osteogenesis, as evident from the expression of important osteogenic markers.

Perhaps the most innovative self‐healable hydrogel for bone tissue engineering is a recently developed protein–DNA hydrogel that can crosslink via DNA hybridization. In this work, a polypeptide copolymer from chemically modified human serum albumin was combined with a rationally designed DNA prepolymer, which could undergo rapid gelation via the addition of a complimentary multiarm DNA cross‐linker. One of the unique features of this protein–DNA hydrogel is its capability to readily assemble bioactive molecules within its structure using properly functionalized DNA adaptors and subsequently release them on demand via the use of a DNA‐cleaving enzyme (DNase). The authors utilized this unique property to custom‐engineer a hydrogel that could remedy the bone resorption caused by osteoclasts in osteoporotic patients through the controlled release of an osteoclast inhibitor. Specifically, the authors reported a significant decrease (≈96%) in the expression of important osteoclast markers, as well as the resorption activity of osteoclast cells. Furthermore, the authors found that the protein–DNA loaded hydrogels did not interfere with the metabolic activity, proliferation or osteogenesis of osteoblast cells.^357^ In our opinion, this protein–DNA hydrogel holds great promise as an agent with the capacity to locally enhance the bone quality in the osteoporotic regions of bone through targeted delivery of bone forming hMSCs and targeted inhibition of osteoclast cell activity.

### 5.2 Cartilage

Articular cartilage lesions are among the most prevalent injuries in the general population and typically caused by trauma, osteoarthritis, or sport‐related injuries.^359^, ^360^, ^361^ Unfortunately, the self‐repair of damaged cartilage is problematic due to the lack of vascularization and slow turnover of its ECM.^362^, ^363^ One avenue for remedying the current situation is through the development of tissue engineering hydrogels to create a biomimetic microenvironment that promotes cell migration, proliferation, and neovascularization. Other properties, such as mechanical strength, engraftment stability, and elasticity, can be further integrated into these hydrogel systems to make them stronger and enable them to better withstand the load‐bearing forces within native cartilage tissue. We envision that tissue engineering hydrogels with sufficient mechanical strength and self‐repair ability can add a much‐needed dimension to the field of cartilage tissue engineering through the manufacture of durable grafts that match the dynamic and load‐bearing microenvironment of native cartilage.

This urgent need for self‐healable cartilage grafts was addressed by Yu et al.^180^ through the development of a novel class of biodegradable, self‐healable, and biocompatible hydrogels based on the integration of dynamic acylhydrazone covalent bonds into an aldehyde‐rich HA‐based hydrogel; as described in detail in Section 3.1.3. Specifically, the aldehyde groups contained in this hydrogel enabled it to adhere to cartilage; as aldehyde groups can bind to the amine groups of local cartilage tissue via Schiff‐base reactions. The adhesive strength between the hydrogel and cartilage was further probed by retrieving native cartilage tissue from porcine, carving a defect into the tissue, pouring the hydrogel into the defect‐site, and performing a push‐out test on the entire composite system. The resistance experienced by the push‐out probe provides a qualitative measure of the adhesiveness between the cartilage and the hydrogel. From these tests, the authors reported an adhesive strength of approximately 10 kPA, which was almost 10‐fold larger than a nonadhesive control hydrogel.

**Figure 16:**
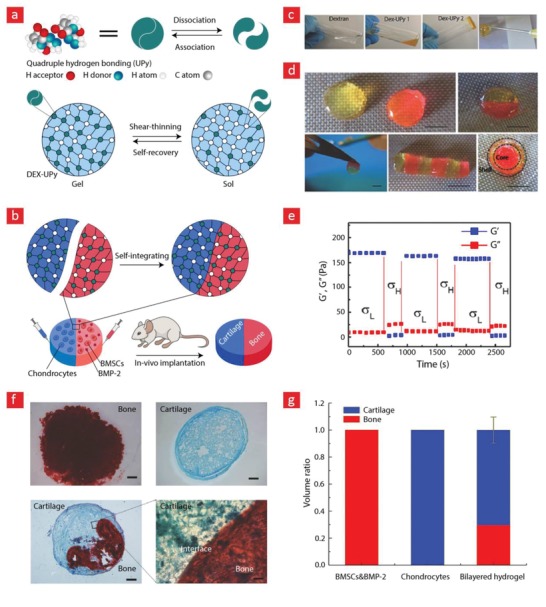
A self‐healable hydrogel for osteochondral engineering made from dextran (DEX) and UPy. a,b) The chemistry behind the self‐healable hydrogel and governing principle behind the formation of osteochondral constructs. c,d) Photographic images showing the hydrogel crosslinking and its self‐healable properties. e) The self‐healing properties were additionally examined through rheology. f) The bone–cartilage interface was stained for bone (Alizarin Red S) and cartilage (Alcian Blue). g) The volume of bone and cartilage after subcutaneous implantation that lasted for 8 weeks. Adapted with permission.^364^ Copyright 2017, Wiley‐VCH.

In another study, dextran was functionalized with Upy to enable the generation of a hydrogel made from reversible hydrogen bonds between adjacent UPy moieties (**Figure** **16**).^364^ To this end, the strong quadruple hydrogen bonding capacity of the UPy moieties endowed the hydrogel with both self‐healing and shear‐thinning properties. The authors emphasized that these properties were strongly affected by the concentration of Upy in the backbone of dextran. The self‐healing capacity of this system was utilized by the authors to merge a pro‐osteogenic hydrogel with a prochondrogenic hydrogel to generate an artificial cartilage–bone interface. This interface was later injected subcutaneously into mice to demonstrate that the construct could mature into an osteochondral‐like graft for furtherer down‐stream tissue engineering applications. Indeed, this concept could open a new paradigm to regenerate arthritis‐infected knees, as one of the devastating consequences of this debilitating disease is the gradual breakdown of the bone‐cartilage (osteochondral) interface.

As mentioned in Section 4, the combination of nanomaterials and self‐healable hydrogels has gained substantial interest in the field due to a host of highly sought‐after properties, such as elasticity, adhesiveness, and bioactivity. Nanoreinforced and self‐healable hydrogels are, therefore, an ideal choice for cartilage tissue engineering, as they can withstand and adapt to the mechanical resilience of native cartilage while also enabling tissue ingrowth. To this end, a recent study demonstrated that incorporation of reversible bonds between calcium phosphate nanoparticles and bisphosphonate‐functionalized hyaluronic acid could facilitate the formation of a highly robust hydrogel with injectable and self‐healing properties. Furthermore, the authors injected the hydrogel into the knee of rats to evaluate its in vivo performance.^365^ The grafts could withstand the highly dynamic environment of the knee for up to 4 weeks and also exhibited some bone formation after 1 week. Although this system is opening new possibilities in cartilage and osteochondral tissue engineering, further efforts should be focused on exploring their stability and in vivo performance to bring this system to the clinic.

### 5.3 Skin

The organs of the human body are not all internal like bone, cartilage, muscle, brain, or the heart. There is one that we wear on the outside of our bodies to protect internal organs, namely skin, the largest organ in the human body. The skin is the body's first line of defense against invasion by pathogens, toxins, or injuries; however, due to its delicate nature, external forces experienced during daily activities easily damage it. Different approaches have been established so far to make the skin healing process faster for patients with acute or chronic wounds. Most of these approaches have focused on the transplantation of skin grafts, such as auto, allo, or xenografts.^366^ Although these strategies have been used clinically and have resulted in promising outcomes for the patients, there are drawbacks associated with the limited number of donors and possible rejection of allo and xenografts.^367^ To remedy this standstill, regenerative wound dressings have in recent years emerged to effectively treat damaged skin tissues.^368^ To this end, different types of wound dressings have been proposed, such as membrane,^369^ rubber,^370^ foam,^371^, ^372^ nanofiber,^373^, ^374^ or hydrogel‐based dressings.^375^ Among these different varieties, there is growing interest in using hydrogel‐based dressings owing to their unique material characteristics. These features include the ability to maintain a moist‐like environment around the wound site, absorb tissue exudates and a high oxygen permeability to facilitate optimal tissue regeneration.^376^ Although hydrogels are ideal candidates for the regeneration of damaged skin, some major challenges still remains unsolved. These challenges mainly include an insufficient mechanical strength to cope with the cyclic movements of human skin, since most hydrogel‐based wound dressing are brittle and unable to heal like natural skin is able to.^377^, ^378^ Therefore, researchers are currently moving toward a new strategy based on tough and self‐healable hydrogel dressings with the capacity to autonomously heal after damage.^377^, ^379^, ^380^, ^381^, ^382^, ^383^, ^384^, ^385^, ^386^ Along these thoughts, Zhao et al.^379^ recently developed an adhesive, conductive, and self‐healable wound dressing by mixing chitosan‐g‐polyaniline (QCSP) with benzaldehyde functionalized poly(ethylene glycol)‐*co*‐poly(glycerol sebacate) (PEGS‐FA).^379^ Notably, this system could crosslink without any external stimuli at 37 °C and, thus, was also injectable. Its self‐healing properties stemmed primarily from dynamic covalent Schiff‐base links between amine groups from QCSP and benzaldehyde groups from PEGS‐FA. In addition to these properties, the positively charged amino groups from the QCSP backbone and the highly anionic nature of polyaniline made the system electrically conductivity. The authors of this study later used their wound dressing on a skin‐defect site in a mouse model to study the in vivo performance of their system (up to 15 d). From these studies, it was shown that use of an OCSP–PEGS–FA dressing leads to a higher expression of wound healing markers (EGF, TGF‐β, and VEGF) as compared to a commercial wound dressing (Tegaderm), which ultimately promoted an almost flawless wound healing process. The authors speculated that these properties were caused by the combination of electrical conductivity and free radical scavenging capacity of the hydrogel, as well as its self‐healing ability, which enabled it to remain intact in the body for 15 d without rupturing. Indeed, several recent studies have demonstrated an interesting link between skin tissue regeneration and electromagnetic fields. Therefore, the system developed by Zhao et al. fits within this exciting and emerging area in the field of skin tissue engineering.^387^, ^388^, ^389^

In other studies, the investigators have relied on polydopamine to develop self‐healable hydrogel‐based wound dressings, as they display formidable adhesiveness to native tissues, excellent biocompatibility, and the ability to endow hydrogels with self‐healing properties due to their highly reactive catechol groups. Some recent studies have tapped into the above‐mentioned portfolio of properties that polydopamine brings by incorporating polydopamine nanoparticles into various hydrogels.^377^, ^380^, ^381^ For instance, polydopamine nanoparticles were recently incorporated into a poly (*N*‐isopropylacrylamide) (PNIPAM) network to generate a promising hydrogel for wound healing applications. This hydrogel displayed exciting properties that included responsiveness to near‐infrared light, self‐healing ability, and tissue adhesiveness.^380^ Specifically, the hydrogel could heal when it was exposed to near‐infrared irradiation and displayed a significantly higher elastic modulus and a greater adhesive strength to porcine skin as compared to pure PNIPAM. The hydrogel also promoted attachment and proliferation of fibroblasts cells, which might be useful for regenerating damaged skin tissues. The regenerative properties of the polydopamine–PNIPAM‐based hydrogel dressing were further tested in an in vivo skin‐defect model in mice, in which a complete wound closure was achieved after 15 d.^380^ In other similar studies, the polydopamine chains were linked to a polyacrylamide network via interactions between free catechol groups and amino groups on polyacrylamide to yield a tough, adhesive, and self‐healable hydrogel‐based wound dressing.^377^, ^390^ This hydrogel could self‐heal after 2 h in an ambient environment without the need of external stimuli, and was also conformable and could adhere to a human arm undergoing twisting and bending due to wrist movements. It could also promote fibroblast adhesion, proliferation, and migration into the wound area, and ultimately the generation of new skin tissue for the treatment of dermal wounds.

In another recent study, it was shown that the combination of dopamine‐modified four‐armed PEG (4‐arm‐PEG‐DA) with phenylboronic acid functionalized four‐armed PEG (4‐arm‐PEG‐PBA) could yield an injectable hydrogel with a good adhesion to skin tissue and rapid self‐healing (30 s).^381^ Especially, the presence of reversible and dynamic phenylborate ester bonds between 4‐arm‐PEG‐DA and 4‐arm‐PEG‐PBA endowed the hydrogels with sufficient self‐healing and mechanical properties and multiple stimuli‐responsive behaviors toward various external stimuli, such as pH, glucose, and dopamine. Most importantly, these hydrogels displayed good cytocompatibility and promoted fibroblast cell attachment. Owing to their rapid self‐healing and exciting adhesion properties, this type of wound dressing provides an exciting opportunity for various wound closure therapies.

### 5.4 Cardiac

Acute myocardial infarction is considering one of the leading causes of mortality worldwide.^391^ Several biomaterial‐based strategies, including epicardial bioengineered patches, and injectable hydrogels, have been developed to prevent heart failure because of postmyocardial infarction.^392^, ^393^, ^394^, ^395^, ^396^ Therapies based on injectable hydrogels are currently rated among the most attractive solutions to deliver therapeutic agents (e.g., drugs or cells) to restore the damaged myocardium because these therapies tend to be highly regenerative while being minimally invasive.^397^ Although hydrogels can retain the delivered cells within a confined region, the pulsatile movements of the heart inevitably lead to stresses that might cause damage and a premature loss of the delivered cellular materials. Self‐healable hydrogels might represent an advantage here; as such, carrier systems can rapidly heal even under high mechanical strains to enable the delivered cells to remain engrafted at the desired location for prolonged periods

In this direction, Bastings and co‐workers have developed an injectable pH‐responsive hydrogel for myocardial drug delivery by using a PEG‐based hydrogel with pH‐sensitive UPy moieties.^398^ The hydrogel is fluid at basic pH (allowing easy injection) but is reversibly transformed into a gel state at neutral pH (when it reaches the heart). Notably, when used as a carrier of growth factors (HGF and IGF‐1), this hydrogel was capable of significantly reducing the size of an infarct scar within a porcine myocardial infarction model as such growth factors enhanced the activation of resident regenerative cells to promote rapid cardiac tissue regeneration.^399^

Another class of self‐healable hydrogels that has been investigated for myocardial repair are based on host–guest interactions between adamantane and beta‐cyclodextrin‐modified HA.^186^, ^187^ The feasibility of using this hydrogel for cardiac therapy has been assessed using an ischemic rat model, in which the stiffness and retention capacity of the hydrogel was significantly enhanced relative to untreated hydrogel controls.^186^ The myocardial function was also remarkably improved when the hydrogel was loaded with endothelial progenitor cells, as the cell‐laden hydrogels caused a significant increase in neovascularization compared to cells delivered alone.^187^

Several recent studies have also shown that scaffold materials displaying electrical conductivity can positively influence the behavior of cardiac cells.^396^, ^400^ Therefore, it could be desirable to use self‐healable hydrogels with electroconductive properties for myocardial repair.^401^, ^402^ To this end, Dong and co‐workers have investigated a self‐healable and electrically active hydrogel that was fabricated by incorporation of aniline tetramer into a dibenzaldehyde‐terminated PEG polymer.^403^ The material displays excellent biocompatibility, as revealed by the viable encapsulation of C2C12 myoblasts and cardiac myocytes, and in vivo cell retention of these cells after subcutaneous implantation. Also, the hydrogel exhibited a fast degradation profile, as subcutaneously injected grafts were completely resorbed over the course of 45 d without inducing a significant inflammatory reaction. Another electroconductive and self‐healable hydrogel for cardiac repair was recently made from the combination of chitosan and polydopamine coated GO nanomaterials.^404^ This hydrogel could crosslink spontaneously by utilizing reversible interactions between catechol groups on adjacent polydopamine polymers and electrostatic interactions between GO and chitosan. Specifically, the GO nanomaterials enabled a simultaneous increase in both the electrical conductivity and mechanical stiffness of the composite due to its multifunctionality. The suitability of this material for cardiac tissue engineering was assessed in vitro using human embryonic stem cell‐derived cardiomyocytes, which exhibited a fast and spontaneous beating rate when combined with the GO‐based hydrogel. Although these proofs of concept studies demonstrate the huge potential of electrically active and self‐healable hydrogels for cardiac repair therapies, the in vivo behavior of this novel class of biomaterials remains to be explored. In particular, it would be relevant to investigate whether these materials could be used as vehicles for simultaneous delivery of active biomolecules and relevant progenitor cells that could efficiently relieve the consequences of acute myocardial infarction.

### 5.5 Neural

Physical insults on the central nervous system (CNS) from car accidents, falls or stab wounds can lead to serious traumatic tissue injuries, which can have devastating consequences for the patients, including lifelong disabilities or even death.^405^ Recent progress in tissue engineering and regenerative medicine has uncovered new therapies for the treatment of such CNS injuries. These approaches involve the use of biomaterial scaffolds to provide a microenvironment that promotes the survival and differentiation of transplanted cells.^406^ Hydrogel‐based scaffolds are ideal for such tissue engineering approaches to target the degenerated CNS as they first reduce the risks of cell damage from the excessive shear stress caused by the injection process, combined with the fact that they present a minimally invasive cell‐engraftment option for the patient.^407^, ^408^

While conventional hydrogels require changes in pH, temperature, or ionic strength to induce a gel phase transition, self‐healable hydrogels are special in the sense that they enable in situ gelation without the need of environmental triggers.^231^ Heilshorn and co‐workers have developed a two‐component hydrogel that exploits the hydrophobic association of peptide‐based domains consisting of hydrophobic amino acids.^409^ This self‐healable biomaterial enabled efficient encapsulation and stable culture of neural‐like PC12 and murine neural stem cells. However, the weak mechanical properties of the hydrogel (shear moduli from 9 to 50 Pa) could hinder its application inside the body, as the shear stress values observed within the human CNS are many times larger.

**Figure 17:**
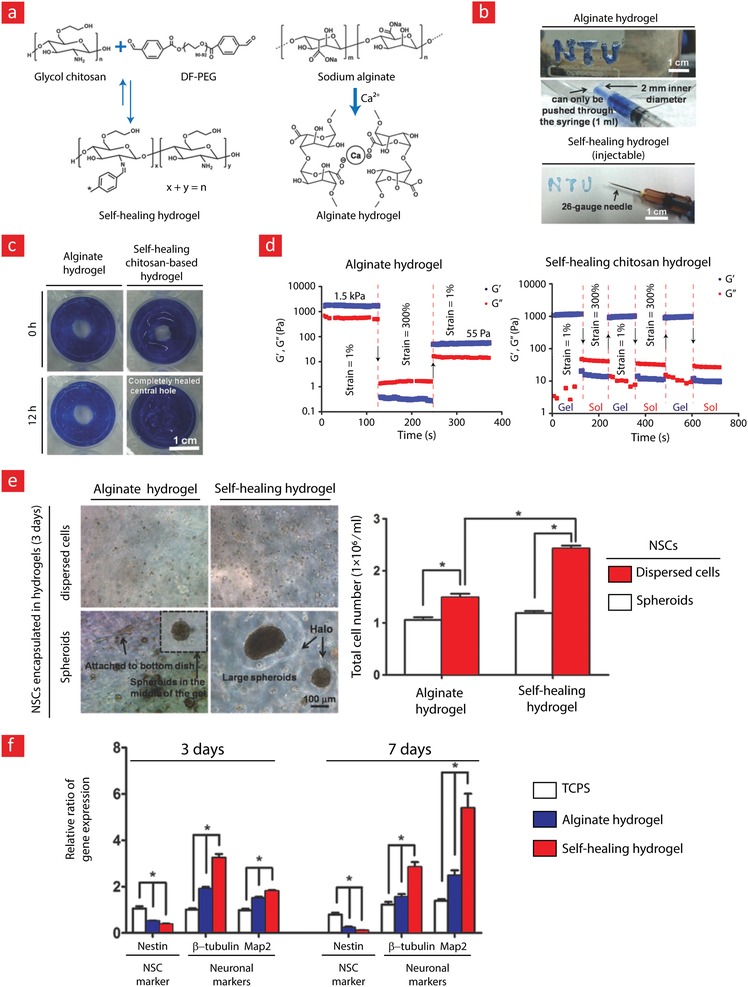
An alginate‐based self‐healable hydrogel for neural tissue engineering. a) The chemistry behind the self‐healable hydrogel. b) Photographic images showing the shear‐thinning properties of the hydrogel. c) Photographic images showing the self‐healing properties of the hydrogel. d) The self‐healing properties of the hydrogel were furtherer quantified through rheology. e) Neural stem cells (NSCs) encapsulated within the hydrogels could from spheroids after only 3 d of culture. f) The expression of important neural markers from the encapsulated NSCs is shown here. Adapted with permission.^40^ Copyright 2015, Wiley‐VCH.

To obtain mechanical properties closer to that of neural tissues, hydrogels comprising amyloid nanofibrils have been suggested as a way to address the current biomaterial challenges in this field.^67^ Notably, these protein‐based hydrogels could support attachment and growth of fibroblasts, neuroblastoma, and mesenchymal stem cells. Other injectable hydrogels that recapitulate the biomechanical properties of the CNS have been manufactured with polysaccharide‐based prepolymers.^40^, ^410^, ^411^ In one of these approaches, Tseng and co‐workers generated a chitosan‐based self‐healable hydrogel with a stiffness of 1.5 kPa—in the same range as the stiffness of neuronal tissues (0.1–1 kPa)—and demonstrated that this system could lead to an enhanced differentiation of encapsulated murine neural progenitors (**Figure** **17**).^40^ Notably, these authors used a zebrafish‐based neural injury model to confirm that only neural spheroids that are encapsulated into hydrogels of appropriate stiffness can significantly heal neural damages.^40^, ^410^ In another approach, a self‐healable hydrogel made from dynamic imine bonds between chitosan and oxidized sodium alginate was developed to carry neural stem cells (NSCs) into the CNS (Figure 17).^411^ In this study, it was shown that it is possible to finely adjust the stiffness of the hydrogel between 100 and 1000 Pa to precisely fine‐tune the growth and differentiation of rat NSCs. The feasibility of this system for CNS cell‐based therapy was also demonstrated by confirming a uniform cell distribution upon injection of the NSC‐loaded hydrogels into mice brains.

Self‐healable hydrogels with tunable electrical properties are also promising candidates for the engineering of neural tissues. As studies have shown, not only mechanical but also electrical cues from the microenvironment are crucial to support the development of neural cells.^412^, ^413^ To this end, Hou and co‐workers have developed an electroconductive graphene‐based hydrogel with self‐healing capacity, which supported the growth of neural‐like PC12 cells.^414^ Although this material appeared to be well‐suited as a substrate for neural growth, its gel‐phase transition occurred under nonphysiological conditions, and thus did not allow 3D encapsulation of cells. This therefore warrants further investigation on self‐healable and electroconductive hydrogels for neural tissue engineering purposes.

In summary, the few seminal in vitro and in vivo studies have demonstrated that it is possible to adjust the structure and biophysical properties of self‐healable hydrogels to control survival, growth, and differentiation of neural cells. Such hydrogels could eventually offer a unique platform for developing injectable stem cell therapies to target the degenerated CNS. However, more work is needed to achieve the potential of these biomaterials as their clinical significance remains to be fully investigated.

### 5.6 Others

**Figure 18:**
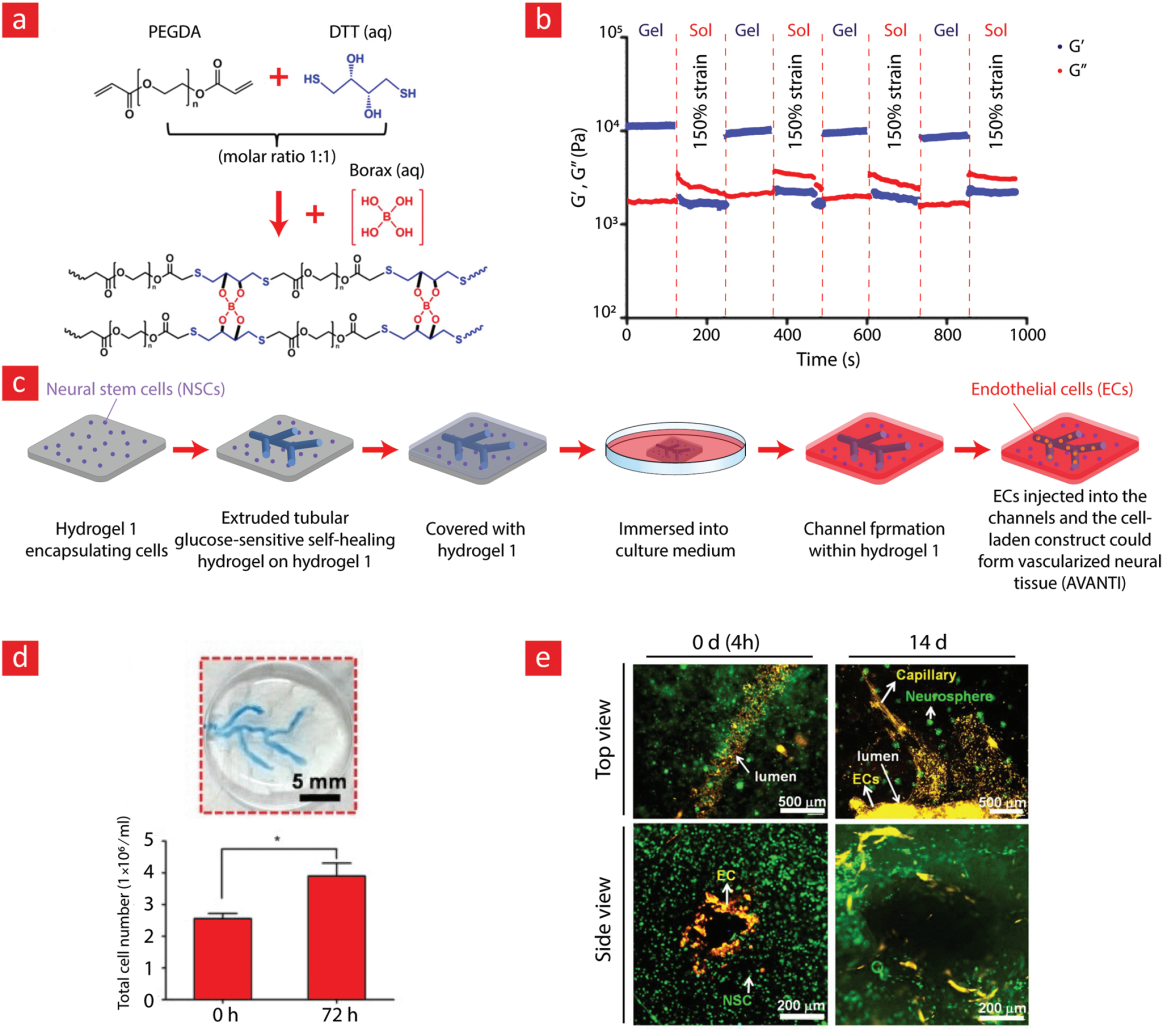
A PEG‐based self‐healable hydrogel for vascularized tissue engineering. a) The chemistry behind the self‐healable hydrogel. b) The self‐healing properties of the hydrogel as quantified through rheology. c) Schematics showing the preparation of the vascularized and self‐healable tissue constructs. d) Photographic images of the tissue engineered construct. e) Fluorescence imaging of the constructs demonstrating the formation of premature lumen‐like structures within neural‐like tissues. Adapted with permission.^421^ Copyright 2017, Elsevier.

In addition to neural, cardiac, bone and cartilage, self‐healing hydrogels have also been utilized to regenerate vascularized,^211^, ^415^ muscle,^113^, ^416^ and gastric tissues.^58^, ^198^, ^417^ For instance, a glucose‐sensitive self‐healing hydrogel based on PEGDA was used as a sacrificial material to generate vascularized constructs (**Figure** **18**).^211^ In this concept, the hydrogel was generated by mixing PEGDA and dithiothreitol followed by the addition of a borax solution to form reversible boronate ester bonds between the individual polymer blocks. The authors speculated that the breaking and reforming of boronate ester bonds were responsible for the self‐healing and glucose‐sensitive properties of this hydrogel. Specifically, these authors fabricated vascularized constructs in a two‐step process, first, self‐healable and microfabricated hydrogels were embedded into a nonglucose‐sensitive fibrin hydrogel. Then, to generate branched tubular channels, the combined hydrogels were immersed in a glucose‐containing culture medium to remove the sacrificial hydrogel from the composite construct. Endothelial cells were then seeded within the channels to facilitate the formation of capillary‐like structures. After 2 weeks of cell culture, a successful generation of 450 µm capillary‐like structures was observed inside a self‐healable hydrogel‐based construct

Self‐healable hydrogel systems have also shown promise for the delivery of stem cells, proteins, drug, and signaling molecules to promote muscle tissue regeneration.^248^, ^416^, ^418^, ^419^ As an example of this feasibility, Mulyasasmita et al. used a peptide/protein modified PEG‐based hydrogel to yield a shear‐thinning and self‐healable hydrogel that could deliver human induced pluripotent stem cell‐derived endothelial cells (hiPSC‐ECs) into ischemic muscle tissues in mice.^416^ This approach resulted in much higher cell retention and better hydrogel graft stability, which ultimately facilitated muscle tissue regeneration within the mouse model. In another study, McKinnon et al.^113^ developed a multi‐arm PEG‐based hydrogel that could crosslink via reversible covalent interactions between hydrazone and aldehyde groups. Notably, this hydrogel could be fine‐tuned to promote proliferation and spreading of C2C12 myoblasts into multinucleated myotubes, which is an important event towards the maturation of myoblast cells into native‐like muscle tissue.

Finally, self‐healing hydrogels have been used as tissue adhesives to seal stomach wounds.^58^, ^417^ In an enlightening study, Phadke et al. demonstrated that, by using a pH‐responsive self‐healable hydrogel [poly (acryloyl‐6‐aminocaproic acid) (PA6ACA)], it is possible to create a stable, elastic, and adhesive hydrogel that can thrive within the highly acidic environment of the stomach, and ultimately close stomach wounds. Specifically, the damaged PA6ACA hydrogel could heal within 2 s upon exposure to an acidic solution (pH ≤ 3) and was thus applicable as an injectable and durable hydrogel for regenerating stomach wounds. In another recent study, a self‐healable carboxymethyl cellulose‐based hydrogel was developed through dynamic ionic coordination interactions between Al^3+^ ions and carboxylate groups (COO^−^) of carboxymethyl cellulose for sealing of stomach wounds.^417^ In vitro studies with fresh gastric tissue from a pig stomach have shown that this hydrogel could adhere well to the gastric mucosa without any external intervention to enable a potentially strong and long‐term sealing of stomach wounds in possible future down‐stream applications.

## 6 Emerging Directions and Future Trends

With the recent advances in self‐healing systems, tissue engineers have the necessary tools to generate electrical conductive and self‐healable hydrogels. The field could therefore easily be expanded into the emerging fields of cyborganics, bioacutators, and injectable bioelectronic hydrogels with shape memory properties. In the following sections, we will give a brief description of these emerging trends and revisit self‐healable hydrogels with electronic properties since they are pivotal in the above‐mentioned applications.

### 6.1 Electronic Hydrogels with Self‐Repair Properties

The field of self‐healable and hydrogel‐based electronics is still in its infancy. However, the emergence of wearable devices, such as electronic skin, google glass, apple watch, and various healthcare monitors, has pushed the field to a new high. In brief, self‐healable electronics encompass electrical conductive materials or circuits that can rapidly self‐repair during wear and tear. When combined with hydrogels and tissues, these could potentially generate self‐healable cyborganics, bioactuators, and injectable soft‐robotic systems for various tissue engineering applications.

**Figure 19:**
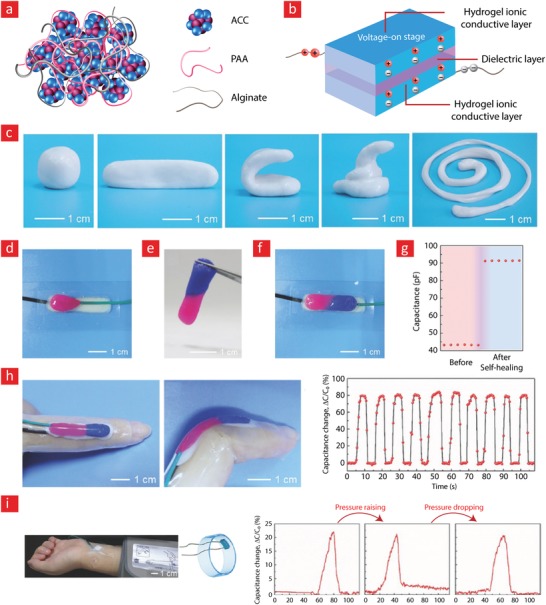
A hydrogel‐based electronic skin with self‐healing properties. a) The chemistry behind the self‐healable hydrogel. b) The working principle behind the developed self‐healable capacitor. c) Photographic images demonstrating the moldability of the hydrogel. d) A photo of the fractured sensor, e) the healed hydrogel, f) and healed hydrogel sensor. g) The capacitance of the sensor before and after the healing process. h) Photographic images of the sensor attached to a finger that is undergoing bending, and the associated change in capacitance during finger movement. i) Real‐time capacitance measurements from a blood‐pressure sensor when the pressure is raised and lowered. Adapted with permission.^421^ Copyright 2017, Wiley‐VCH.

As briefly mentioned in Section 4, the easiest way to develop hydrogel systems that are both self‐healable and electrically active at the same time is through nanoreinforcement with carbon‐based materials such as graphene or carbon nanotubes. Indeed, several recent studies have demonstrated that this pathway can lead to some exciting electronic devices. Examples include a range of hydrogels that were incorporated with carbon‐nanotubes,^314^, ^420^ calcium carbonate nanoparticles,^421^ clay nanoparticles,^252^, ^390^ ferric ions,^87^, ^422^, ^423^, ^424^ or graphene^47^, ^425^ to yield self‐healable bioelectronics, bioactuators, and electronic skin (e‐skin) devices. For instance, in a recent study Lei et al.^421^ embedded calcium carbonate nanoparticles into an alginate–polyacrylic acid hydrogel to generate an ionic conductor (**Figure** **19**). As briefly highlighted in Section 4.2, such systems exhibit self‐repair properties due to the ionic interactions between Ca^2+^ and the negatively charged chains of alginate and polyacrylic acid. The Ca^2+^ ions also gave the system its ionic conductivity, and by integrating these ionic conductors with a dielectric layer it was possible to generate a self‐healable capacitor. As the capacitance is intimately linked with the area of the capacitor, any deformation experienced by such devices will immediately result in measurable capacitance changes. The device was therefore used for human motion detection and blood pressure measurements through area‐facilitated changes in the capacitance. Interestingly, because the ionic conductor itself was self‐healable this device also displayed self‐healing properties and mimicked many of the fascinating properties such as self‐repair, elasticity, and deformability of natural skin. In a similar study, poly(vinyl alcohol) was functionalized with *N,N,N*‐trimethyl‐1‐(oxiran‐2‐yl)methanaminium chloride to yield a self‐healable hydrogel in the presence of borax via dynamic diol–borate ester bonds.^426^ In addition, a KCL solution was incorporated into the hydrogel to generate a self‐healable electrolyte; likewise activated carbon and acetylene black was embedded within the hydrogel to yield self‐healable electrodes. The sandwiching of the self‐healable electrolyte between the electrodes therefore resulted in the formation of a self‐healable capacitor with the self‐healing mechanism being governed by diol–borate ester bonds. Amazingly, the device could retain up to 84.8% of its capacitance after bending for up to 1000 times. The device was also able to completely heal after 5 min and sustain its current‐voltage profile after 15 cycles. It therefore has the potential to transform into a self‐healable and wearable e‐skin device by using the same method reported in the recent study by Lei et al.^421^

A series of milestone contributions in the field has also lead to polymer‐based lithium batteries^423^, ^427^ and semiconductors^300^, ^428^ with self‐repair capacity. Notably, in a recent study, carboxymethylcellulose and aligned CNT sheets were combined with one another to yield an electrically active self‐healable hydrogel.^427^ The self‐healing mechanism was mediated by hydrogen bonds between the cellulose fibers and weak Van der Waal bonds among the CNTs. By loading this hydrogel with LiMn_2_O_4_ and LiTi_2_(PO_4_)_3,_ it was possible to generate a self‐healable and flexible lithium battery for wearable healthcare monitoring. Specifically, it was demonstrated that the device could retain almost 90% of its capacitance after 200 bending cycles, heal instantly, and maintain 92% of its mechanical properties after five cutting‐healing cycles.^427^

Despite the significant progress in the field of self‐healable and hydrogel‐based electronics none of these systems really incorporated complicated electronic circuitries, which is a requirement for cyborganic, bioactuating, and soft‐robotic devices. One avenue around this bottleneck is through the microfabrication of healable circuits based on carbon nanotubes or liquid metals. These circuits can then become encapsulated within a hydrogel and conjugated to the hydrogel backbone through various healable links. In the following sections, we will look into such future applications and briefly discuss their implications for the field of tissue engineering.

### 6.2 Cyborganics

The term cyborg tissues/organs first emerged in 2012 in a series of news articles to define the possible merger between artificially grown tissues and inanimate nanomaterials.^429^ Such cyborg organic constructs (cyborganics) utilize the recent technological advances in the field of materials science, chemistry, electronics, and tissue engineering to yield tissues that are half‐man and half‐synthetic. These can be divided into two levels based on their level of complexity; those made from simple reinforcement of artificially engineered tissues with nanomaterials;^93^, ^430^, ^431^, ^432^, ^433^ while the “level twos” are mergers between artificial tissues and more complex matters such as electronic circuits, robotic systems, and intelligent materials.^434^, ^435^, ^436^, ^437^, ^438^, ^439^ Being the most “easy‐ones‐to‐manufacture” the level ones have been rapidly picked up by the field to give rise to a series of milestone contributions. On the other hand, the “level twos” have not soared as high as the level ones and therefore needs much future attention to reach the same level of maturity. An important gateway into this almost “sci.fi‐like” future was opened with a number of earth shattering publications from Charles Liebers research group.^434^, ^437^, ^438^, ^439^ Especially, one study has truly opened up the realm of real‐world applications for level two cyborganics.^439^ In this study,^439^ intricate silicon‐based nanocircuits were intertwined with living cardiac tissues to generate something “out‐of‐this‐world”; something that could potentially in situ monitor the irregular beating of dysfunctional cardiac cells, heart inflammation, and potentially dangerous microenvironmental pH changes.

In the same spirit as the futuristic studies from Liebers group, we will in the following paragraphs proposed a feasible roadmap for the manufacture of self‐healable cyborganics based on carbon‐based nanomaterials. The key here is the possibility of reversible π–π interactions between neighboring CNT's or RGO sheets, which we anticipate will enable the formation of highly conductive and self‐healable links. For instance in a recent study it was shown that the freeze‐drying of reduced graphene could lead to complex conductive structures that were held together by reversible π–π interactions.^314^, ^341^ Such high‐order structures could easily be embedded within self‐healable hydrogels for further down‐stream cell culturing to yield cyborganics with the capacity to spontaneously heal as natural tissues do. Another interesting development is liquid metal circuits as they are both easy to mold and self‐healable^440^ Again, like the other ones we also anticipate that such circuits are readily embeddable within various self‐healable hydrogels.

Self‐healable cyborganics will likely facilitate a breakthrough that could potentially reform society into a more versatile form, wherein individuals fully master their health through wireless monitoring. For instance, the expansion of the field into controllable and self‐healable cyborganics will enable mankind not only to transcend his own biology beyond its current limitations but also enable him to control when and how his body heals. As the field advances, we also anticipate a number of unanswered ethical questions, that needs to be carefully addressed before the gates into the realm of cyborganics can fully open as this field is per say a highly controversial one.

### 6.3 Bioactuators

Bioactuators are devices that use the combined powers of biology and materials science to generate life‐like systems that can move, crawl, swim, sense, and assist in important tissue functions.^441^, ^442^ They have been explored in a wide range of applications such as various robotic systems in the industry,^442^ tissue engineering^93^ and smart drug delivery systems.^441^ Bioactuators are typically made from a combination of locomotive cells derived from muscle and cardiac tissues, and conductive polymers that can contract and expand in an almost inexhaustible manner. As cells within locomotive tissues typically form anisotropic structures, one of the key principles behind cardiac and muscle‐based bioactuators is the alignment of cells into linear microstructures. Examples include, the usage of cardiac cells that were genetically engineered to respond to light and aligned within a stingray‐like elastomeric body to yield a stingray‐like creature capable of controlled locomotion within a light field;^443^ and a bioactuator consisting of CNT's aligned within GelMA to generate a more native‐like electrical coupling between encapsulated cardiomyocytes to enable a more coherent bioactuation in physiological conditions.^444^

Bioactuators possesses a great deal of potential and if used properly they can open up for new groundbreaking opportunities in the defense sector, industry, healthcare system, and maybe even in the private sphere; who knows the end of the road? One thing is certain, though: the opportunities as we speak seem limitless and the field has established itself as one with the capacity to create a series of landmark changes in society. Even still, in the author's opinion the best is yet to come in the form of self‐healable bioactuators with the ability to spontaneously heal in demanding scenarios such as in the battlefield, hazardous, and dangerous working environments and within the sometimes highly strenuous milieus of the body.

**Figure 20:**
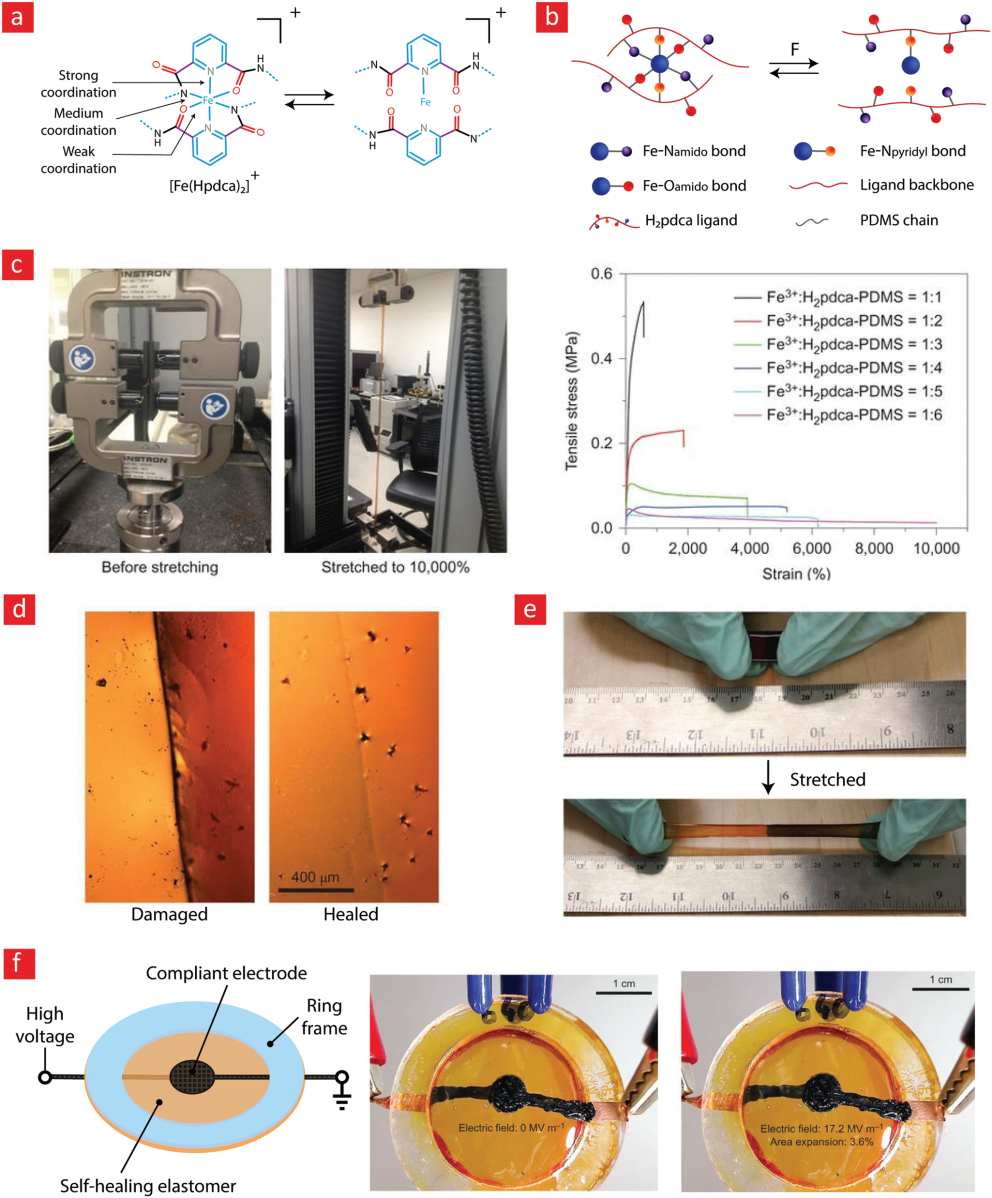
A bioactuator with self‐healing properties. a,b) The self‐healing chemistry behind the generated device. c) Tensile measurements of the device demonstrated a stretchability that could reach 10 000%. d,e) Photographic images demonstrating the self‐healing properties of the device. f) The self‐healable device could actuate in response to an alternating electrical field. Adapted with permission.^423^ Copyright 2012, Macmillan Publishers Ltd.

Along these thoughts, a recent study from Stanford University showed that a self‐healable bioactutaor could be made from the combination of a Fe(III)‐2,6‐pyridinedicarboxamide (PDCA) chelation complex and poly(dimethylsiloxane) (PDMS) polymer (**Figure** **20**).^423^ The PDCA–PDMS system broke the elasticity record for elastomers by stretching to 10000% its original length without breaking. This system also displayed autonomous self‐healing ability, as it was able to almost completely self‐heal after 48 h at room temperature. In addition to the self‐repair ability and high stretchability, the PDCA–PDMS system displayed interesting electrical properties because of the iron ions, which enabled it to actuate with ease during electrical stimuli. The authors of this study speculated that the native‐like actuating properties of the PDCA–PDMS system could potentially be used to manufacture artificial muscles for various self‐healable soft robotic systems. In our opinion, the incorporation and alignment of muscle cells within the PDCA–elastomers could make its bioactuating properties more human‐like, making it more amenable to human–machine interface applications.

Building on the exciting results from the Stanford study^423^ it would be interesting to also incorporate complex electronic structures into the PDCA–elastomer to develop more sophisticated actuators with self‐repair properties. Indeed, several methods are already available for the incorporation of self‐healable liquid metal circuits into PDMS‐based elastomers.^440^ The most frequently used one is based on the generation of microfluidic channels within PDMS followed by microfluidic injection of liquid metal. Due to the shear‐thinning properties of most liquid metals, the metal will readily flow into such microchannels and form well‐controlled electronic circuits for further downstream applications.^445^

**Figure 21:**
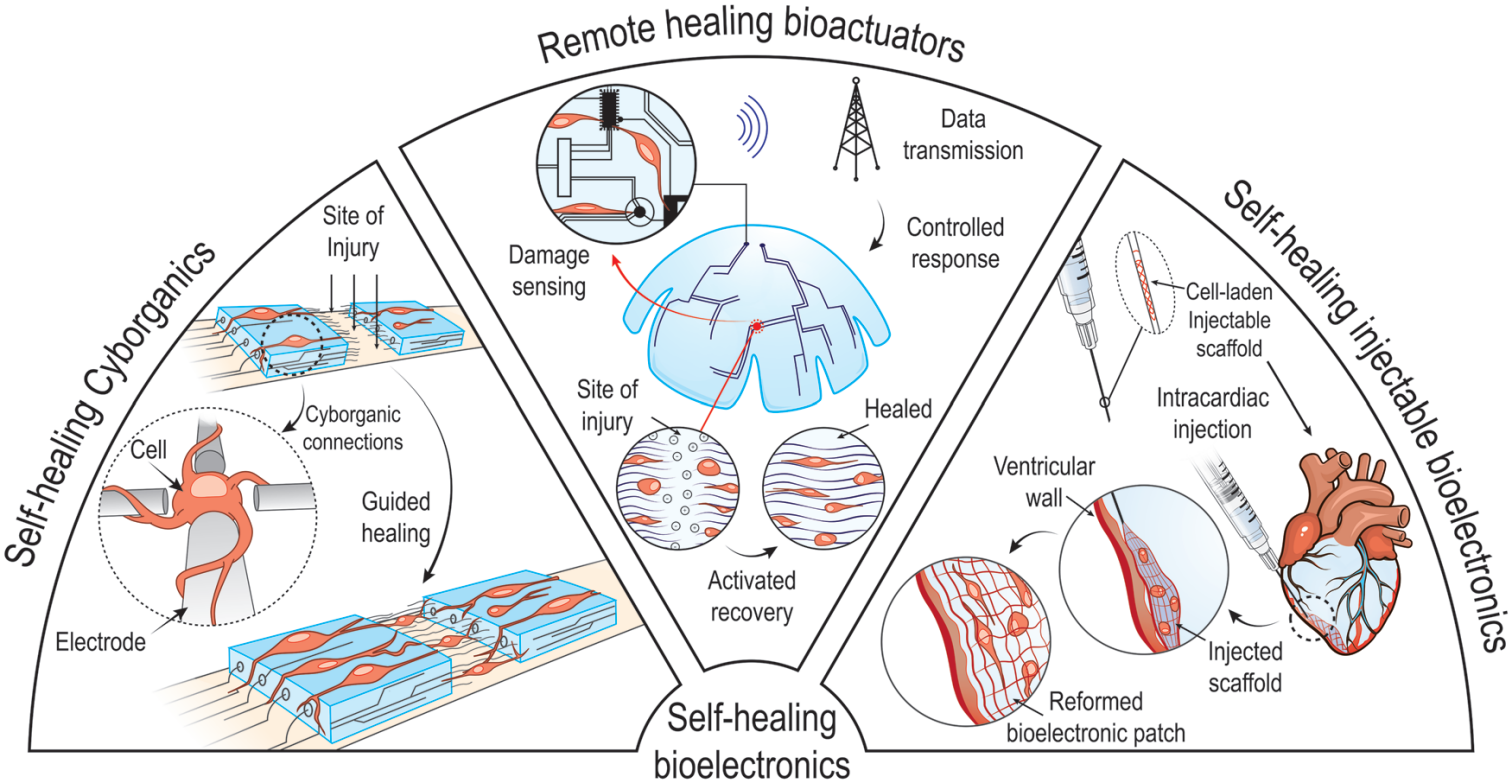
Emerging trends and future directions in the field.

With regard to future research directions, special attention should be given to the merger of bioactuators and cyborganics (**Figure** **21**). In our opinion, such hybrid creatures can use the working principle behind cyborganics to become more powerful soft robots; as well as being healed from a distance through remote controlling. The prospect of such self‐healable android‐like robots is certainly of huge interest for governments worldwide, being potentially useful for militaries preparing for the challenges of the next century and potentially enabling humans to journey to hard to reach and inhabitable places on Earth.

### 6.4 Electronic Tissue Engineering Hydrogels with Shape‐ Memory Properties

In recent years, injectable hydrogels have emerged as a promising alternative to minimize the many risks and complications associated with surgical implantation of tissue engineering scaffolds.^446^ Especially, hydrogels that can completely restore their geometric properties after needle injection through a small‐bore needle have garnered a huge interest in the field, as they enable delicate native‐like architectures to fully survive the injection phase.^447^, ^448^ For electroactive tissues, it is also desirable to incorporate certain electronic circuits within the tissue engineering scaffold to both improve the performance of the scaffold and enable remote monitoring of what is going on in the target tissue. Of course, the many delicate features of such circuits will not survive the injection phase under normal circumstance and, therefore, need to encompass materials that can self‐restore within the body. The research group of Charles Lieber has pioneered the development of electronic circuits that can survive the injection phase into the body.^438^, ^449^, ^450^ To this end, Charlies Lieber's group used flexible and mesh‐like electronics that can fold and unfold many times, while keeping their electrical properties intact. We believe that it would be interesting to incorporate such mesh‐like electronics into self‐healable hydrogels with shape memory properties to develop injectable cyborganics, cardiac patches, or artificial muscle actuators that have sufficient durability for proper performance within the load‐bearing and dynamic tissues of the body.

## 7 Conclusion

Self‐healable hydrogels have been rapidly adopted by tissue engineers as a new class of biomaterials with the potential to push the field of tissue engineering to new heights. The union between hydrogel toughness and self‐healability, as well as the multifunctional properties of self‐healable and nanoreinforced hydrogels, have been thoroughly discussed in this review, as we believe these systems will reshape the field. Especially, the nanoreinforced hydrogels present a new avenue in the field that could potentially be exploited to yield self‐healable electronic hydrogels, cyborganics and soft biorobots. We believe that these systems encompass some exciting concepts that will push the field of biomedical engineering to a new high in the coming decades.

Although the above‐mentioned hydrogels hold great promise, the design and development of such systems pose several challenges that need to be addressed to enable the regeneration of damaged tissues and the manufacture of cybernetic devices that are partly living and partly machine. To this end, it is important to control the biodegradability of the hydrogels to both enable tissue ingrowth and cell migration, but also, to enable the hydrogels to thrive in physiological environments. Otherwise, they might degrade too quickly and jeopardize the long‐term performance of the devices within the human body.

The tissue engineering hydrogels also need to promote sufficient cell spreading, proliferation, and differentiation. Otherwise, their regenerative performance within the body would be significantly compromised. Also, many of the described systems in this review paper have not yet been tested in animals, and their biocompatibility in the human body remains an open question. We anticipate that the recently intensified synergy between the fields of medicine, physics, chemistry, nanoscience, biology, and mechanical engineering can address some of these issues. In our opinion, a combined effort from these cross‐disciplinary fields is needed to develop nontoxic (chemistry), mechanically strong (mechanical engineering), electronic (physics), and clinically relevant hydrogels. Such interdisciplinary collaborations will undoubtedly contribute to bridging the current gaps between the laboratory and the clinic.

## Conflict of Interest

The authors declare no conflict of interest.
